# Gene Expression Analysis of HPRT-Deficient Cells Maintained with Physiological Levels of Folic Acid

**DOI:** 10.3390/cells14141105

**Published:** 2025-07-18

**Authors:** Rosa J. Torres, Gerard Valentines-Casas, Claudia Cano-Estrada, Neus Ontiveros, José M. López

**Affiliations:** 1 Department of Biochemistry, Hospital La Paz Institute for Health Research (IdiPaz), 28046 Madrid, Spain; 2 Center for Biomedical Network Research on Rare Diseases (CIBERER), ISCIII, 28006 Madrid, Spain; 3 Institut de Neurociències, Universitat Autònoma de Barcelona, 08193 Cerdanyola del Vallès, Barcelona, Spain; gerard.valentines@autonoma.cat (G.V.-C.); claudia.cano@uab.cat (C.C.-E.); neus.ontiveros@uab.cat (N.O.); josemanuel.lopez@uab.cat (J.M.L.); 4 Unitat de Bioquímica, Departament de Bioquímica i Biologia Molecular, Facultat de Medicina, Universitat Autònoma de Barcelona, 08193 Cerdanyola del Vallès, Barcelona, Spain

**Keywords:** Lesch–Nyhan, HPRT, nervous system development, RNAseq, purine

## Abstract

Lesch–Nyhan disease (LND) is associated with a complete deficiency of hypoxanthine-guanine phosphoribosyltransferase (HPRT) activity due to mutations in the HPRT1 gene. Although the physiopathology of LND-related neurological manifestations remains unknown, a defective neuronal developmental process is the most widely accepted hypothesis. We generated an HPRT-deficient line from the pluripotent human embryonic cell line NT2/D1 by CRISPR-Cas9 and induced its differentiation along neuroectodermal lineages by retinoic acid treatment. As levels of folic acid in the culture media may affect results in LND models, we employed physiological levels of folate. The effect of HPRT deficiency on neural development-related gene expression was evaluated using two methodological approaches: a directed qPCR array of genes related to neuronal differentiation, and global gene expression by RNAseq. HPRT-deficient pluripotent cells presented altered expression of genes related to pluripotency in human embryonic stem cells, such as *DPPA3* and *CFAP95*, along with genes of the homeobox gene family. HPRT-deficient pluripotent cells were able to differentiate along neuro-ectodermal lineages but presented consistent dysregulation of several genes from the homeobox gene family, including *EN1* and *LMX1A*. GO enrichment analysis of up- and downregulated genes in HPRT-deficient cells showed that the most significant biological processes affected are related to development and nervous system development.

## 1. Introduction

Lesch–Nyhan syndrome (OMIM 300322) or Lesch–Nyhan disease (LND) is an inborn error of purine metabolism characterized by hyperuricemia and hyperuricosuria, severe action dystonia, choreoathetosis, ballismus, macrocytosis, cognitive and attention deficit, and aggressive and self-injurious behavior [1,2]. The disease is associated with a complete deficiency of hypoxanthine-guanine phosphoribosyltransferase (HPRT) activity due to mutations in the *HPRT1* gene [3]. This gene is located in the long arm of the X chromosome, and HPRT deficiency is inherited as a recessive X-linked trait, thus meaning that males are affected and women are generally asymptomatic carriers [4]. Less-severe clinical presentations are suspected to be due to partial enzyme deficiency [5]. It is considered that a continuous spectrum of neurological involvement is present in HPRT-deficient patients, and the term Lesch–Nyhan attenuated variants (LNV) has been introduced to include patients with HPRT-related gout and variable degrees of neurological involvement but without the complete LND phenotype [6].

HPRT catalyzes the salvage synthesis of inosine monophosphate (IMP) and guanosine monophosphate (GMP) from the purine bases hypoxanthine and guanine, respectively, using 5′-phosphoribosyl-1-pyrophosphate (PRPP) as a co-substrate. HPRT deficiency results in the accumulation of its substrates, hypoxanthine, guanine, and PRPP. Hypoxanthine is converted into uric acid by means of xanthine oxidase, and the de novo purine synthesis is increased due to a greater availability of PRPP for PRPP amidotransferase, the rate-limiting enzyme in the pathway, as well as the decrease in IMP and GMP, which are PRPP amidotransferase feedback inhibitors. This dual mechanism results in hugely increased uric acid production [7]. Uric acid overproduction is present in all HPRT-deficient patients, and its severity is not related to the grade of enzymatic deficit. Without treatment, it is manifested as renal lithiasis and gout. Acute renal failure, due to obstructive lithiasis, may be the first sign of the disease. Arthritis due to urate monosodium deposition and tophi may develop later, although nowadays they are rare due to early treatment. Prior studies have demonstrated the transfer of purines through the hematoencephalic barrier, and, as a consequence of HPRT deficiency, there are increased levels of both hypoxanthine and 5-aminoimidazole-4-carboxamide riboside (AICAr) in the cerebrospinal fluid and urine of LND patients [8]. AICAr is a nucleoside derived from AICAR, also known as ZMP, an intermediate of the de novo purine biosynthetic pathway. Toxic effects of these metabolites have been postulated in the pathogenesis of neurological dysfunction [9,10]. However, the connection between aberrant purine metabolism and the neurological, hematological, and behavioral characteristics of the disease remains unknown. Studies with positron-emission tomography and ligands that bind to dopamine-related proteins suggest an alteration of the dopaminergic system in LND patients [11,12]. Furthermore, dopamine deficiency in the striatum has also been confirmed in knockdown mouse models of HPRT deficiency, although these models do not reproduce the manifestations of human disease [13]. Recently, it has been described that HPRT1 knockout mice have neurodevelopmental alterations during embryogenesis, affecting the proliferation and migration of midbrain dopaminergic neurons [14]. Although conventional imaging studies and post-mortem examination of brains from LND patients have not revealed any characteristic morphological abnormality, a reduction of about 20% in intracranial and brain volumes has been found in LND patients by voxel-based morphometry [15]. Moreover, although clinical data suggest a basal ganglia alteration, brain volume abnormalities in grey and white matter in LND are not restricted to the basal ganglia, showing that the effects of HPRT deficiency are probably not limited to the nigrostriatal dopamine pathways [15,16]. The most widely accepted hypothesis at this time is that a defective neuronal developmental process is implicated in LND-related neurological manifestations, rather than having a degenerative cause. As animal models have failed to reproduce human disease, several culture models, using human or mouse cells, have been employed to study neuronal development in HPRT deficiency. These models include induced pluripotent stem cells (iPSCs) derived from fibroblasts from LND patients, neural stem cells (NSCs) prepared from iPSC cells, and NSCs obtained from LND fetuses, among other cellular models [17,18,19,20,21]. A pioneering study [22] indicated that HPRT could regulate the early developmental program of dopamine neurons, and, subsequently, other studies confirmed that HPRT-deficient cells presented some alterations in transcription factors related to neural development [23,24,25]. However, although a decrease in average neurite length has been detected in human [20,23] and mouse [26,27] cell models, other studies showed no obvious differences in morphological appearances [17,18,19,28] or overall growth rates in NSCs HPRT-deficient cells. In summary, although some abnormalities have been detected in the early neural development stage, certain discrepancies have been observed that could be due to the different cellular models and culture media used.

We have previously found that purine alterations in LND fibroblasts depend significantly on the level of folic acid in the culture medium [8,29]. In humans, the de novo purine synthesis requires N10-formyltetrahydrofolate as a cofactor. In HPRT deficiency, there is a significant acceleration of the de novo purine biosynthetic pathway, and under these circumstances, physiological folate levels may become limiting, inducing the accumulation of ZMP in LND fibroblasts [8,29].

In light of the above, the aim of this study was to evaluate the effect of HPRT deficiency on the expression of genes related to neural development using a proper cell model cultured at physiological levels of folic acid. NT2/D1 is a pluripotent human testicular embryonal carcinoma cell line that differentiates along neuro-ectodermal lineages after exposure to retinoic acid (RA) [30,31] and is a widely accepted model for the study of neuronal differentiation. To evaluate the effect of HPRT deficiency on the expression of genes related to neural development, we employed two methodologies: Real-time quantitative PCR of selected genes related to neuronal differentiation and differential global gene expression by RNAseq analysis.

## 2. Materials and Methods

### 2.1. Generation of Pluripotent HPRT-Deficient Cells

We employed the pluripotent human embryonic cell line NTERA-2 cl.D1 (NT2/D1) (American Type Culture Collection [ATCC] ^®^ CRL1973™, Manassas, VA, USA), a pluripotent human testicular embryonal carcinoma cell line. We obtained a pool of NT2/D1 CRISPR-Cas9 HPRT1 gene-edited cells from Synthego (Synthego Corporation, Redwood City, CA, USA) with at least a 50% HPRT1 gene knockout. This pool contains a heterogeneous mix of cells, some unedited and some with different genetic alterations at the *HPRT1* targeted locus. In order to produce a cell line with complete *HPRT1* knockout, CRISPR-Cas9 edited cells were cultured in regular medium (see composition below) containing 30 µM 6-thioguanine, which is a guanine analogue that is readily converted to thioguanine monophosphate (TGMP) by HPRT. High levels of TGMP and its nucleotide triphosphates TGTP and dTGTP are toxic to cells, and, therefore, HPRT-deficient cells are positively selected against wild-type cells.

### 2.2. Cell Culture Medium and Differentiation of NT2/D1 

Both wild-type and 6-thioguanine-resistant HPRT-deficient NT2/D1 cells were maintained in Dulbecco’s modified Eagle’s medium (DMEM) without folic acid (D2429, Sigma-Aldrich, Saint Louis, MO, USA) supplemented with 9062 nM folic acid (regular medium) or 50 nM folic acid (physiological conditions), sodium bicarbonate 1.5 g/L, D-glucose 4.5 g/L, and L-glutamine 2 mM, and 10% (*v*/*v*) fetal bovine serum (FBS) (ATCC, Manassas, VA, USA.),) and incubated at 37 °C in a humid atmosphere of 5% CO_2_. For gene expression experiments, cells were maintained with physiological levels of folic acid (50 nM) for at least one week and dislodged from the flask by trypsine-EDTA (Sigma-Aldrich, Saint Louis, MO, USA) treatment, aspirated, and centrifuged at 250 g into pellets. The cell pellet was washed in PBS (Gibco), and total RNA isolated using the PureLink RNA kit (Ambion, Austin, TX, USA). For retinoic acid (RA)-induced differentiation, 10 μM RA (R2625, Sigma-Aldrich, Saint Louis, MO, USA) was added to the culture medium containing 50 nM folic acid, and the cells were maintained for 4 weeks, as described by Pleasure et al. [30]. The culture medium was changed three times a week, and at the end of the four-week period, total RNA was isolated as described previously.

### 2.3. 6-Thioguanine Toxicity Assay

CRISPR-Cas9 HPRT1 gene-edited cells were cultured in DMEM regular medium (described above) containing 9062 nM folic acid and 30 µM 6-thioguanine for 10 days. On days 0, 3, and 9, cells were fixed with 4% paraformaldehyde and stained with crystal violet. Images were taken from fixed cells at 20× magnification using the EVOS Floid Imaging System (Thermo Fisher, 4471136, Waltham, MA, USA). After washing four times with PBS, cells were dried and dissolved in methanol to measure absorbance at 570 nm.

### 2.4. DNA Sequencing

DNA and total RNA were obtained from wild-type and 6-thioguanine-resistant cells. *HPRT1* gene exons were amplified by PCR and sequenced [32].

### 2.5. HPRT1 Expression Levels and HPRT Activity

For quantification of *HPRT1* mRNA, 1 μg of total RNA was reverse-transcribed into a first-strand cDNA template using ImProm-II™ Reverse Transcriptase (Promega, Madison, WI, USA). *HPRT1* mRNA levels were determined by real-time PCR employing the β-Actin (ACTB) housekeeping gene for normalization purposes, as described previously [32]. HPRT activity was determined in cell lysates as described by Rylance et al. [33].

### 2.6. Hypoxanthine and Xanthine Determination

Medium from confluent cultures was obtained to measure hypoxanthine and xanthine concentration by the HPLC method, as described previously [34]. Total protein concentration in the cell lysate was measured with Coomassie Protein Assay Reagent (Thermo Scientific, Waltham, MA, USA).

### 2.7. Western Blotting

Total cell lysate was obtained by the addition of 250 µL lysis buffer A (50 mM Tris, 2% SDS, pH 6.8) to one 100 mm dish of cultured cells and heating at 100 °C for 5 min. The samples collected (50 µg protein) were denatured in Laemmli sample buffer at 100 °C for 5 min. Electrophoretic separation was performed on a 10% polyacrylamide gel, and proteins were transferred to a PVDF membrane in transfer buffer (25 mM Tris, 192 mM glycine, 10% methanol) at 90 V for 90 min. After transfer, the membrane was washed with TBST (25 mM Tris, 135 mM NaCl, pH 7.5 and 0.1% Tween20) and blocked for 60 min with 5% dried skimmed milk in TBST, washed several times with TBST, and then incubated overnight at 4 °C with primary antibodies for HPRT (Santa Cruz, Dallas, TX, USA, sc-376938; 1:1000), and β-actin (Sigma-Aldrich, Saint Louis, MO, USA, A1978; 1:500), diluted in TBST containing 5% BSA (Sigma-Aldrich, Saint Louis, MO, USA, A9647). Antibody binding was detected the next morning by washing the membrane with TBST several times and incubating for 1 h at room temperature with horseradish peroxidase-coupled (HRP) secondary antibody (anti-rabbit HRP from Invitrogen, Carlsbad, CA, USA, 31460; or anti-mouse HRP from BD Bioscience, San Jose, CA, USA, 554002) diluted 1:3.000 in TBST containing 5% dried skimmed milk. The membrane was washed several times with TBST, and the signal was detected by chemiluminescence in a Biorad Chemidoc (Bio-rad, Hercules, CA, USA).

### 2.8. Real-Time Quantitative PCR Array of Selected Genes Related to Neuronal Differentiation in HPRT-Deficient and Wild-Type NTD2/D1 Cells

Through a search of recent literature describing gene expression changes during neuronal differentiation, we selected a total of 42 genes related to neuronal differentiation, including Wnt/β-catenin, transforming growth factor beta (TGFβ), and sonic hedgehog (SHH) pathways, to design a qPCR array. The qPCR panel also includes the *HPRT1* gene and four housekeeping genes for normalization purposes (Supplementary Table S1). To quantify gene expression of selected genes, 1 μg of total RNA was reverse-transcribed into a first-strand cDNA template using ImProm-II™ Reverse Transcriptase (Promega, Madison, WI, USA). Real-time PCR was performed employing a designed qPCR array in a Roche LightCycler 480 system using Perfect Probe SYBR Green PCR Master Mix (AnyGene^®,^ Paris, France) with reverse transcription cDNA diluted to 1/12 as a template. An adequate intron-spanning PCR assay was designed for all genes, and lyophilized primers were obtained in a 96-plate format (SignArrays^®^ 96 plates, AnyGene^®^, Paris, France). Expression was quantified in a Roche LightCycler (Roche Diagnostic GmbH, Mannheim, Germany) using a relative quantification method. The expression variation (EV) analysis, based on the “delta delta Cp” (ΔΔCp) calculation method, allows a comparison between different experimental conditions, after normalizing the gene expression results with the selected reference genes (*ACTB*, *RPLPO*, *GUSB*, and *TBP*). A Student’s *t*-test for 2(−ΔΔCp) values was performed comparing the experimental condition and the control sample, and values with a cutoff EV ≥ 2.0 and *p* < 0.05 were considered significant.

### 2.9. Differential Global Gene Expression by RNAseq in HPRT-Deficient and Wild-Type NTD2/D1 Cells

RNAseq analyses were performed by Arraystar Inc. Briefly, mRNA was isolated from 1 to 2 µg total RNA using oligo-(dT) magnetic beads (NEBNext^®^ Poly(A) mRNA Magnetic Isolation Module), followed by RNA-seq library preparation using KAPA Stranded RNA-Seq Library Prep Kit (Roche Diagnostic GmbH, Mannheim, Germany). The completed libraries were qualified using an Agilent 2100 Bioanalyzer and quantified by the absolute quantification qPCR method. For sequencing, the barcoded libraries were mixed, denatured to single-stranded DNA in NaOH, captured on an Illumina flow cell, amplified in situ, and subsequently sequenced for 150 cycles for both ends on the Illumina NovaSeq 6000 instrument. Image analysis and base calling were performed using the Solexa pipeline v1.8 (Off-Line Base Caller software, v1.8). Sequence quality was examined using the FastQC v1.0.0 software [35]. The trimmed reads [36] were aligned to the reference genome using Hisat2 v2.0.4 software [37]. The transcript abundances for each sample were estimated using String Tie [38,39]. The fragments per kilo base of transcript per million mapped reads (FPKM) values for gene and transcript level were calculated using the R package Ballgown v2.6.0 [40]. Differentially expressed gene analyses were performed with the R package Ballgown v2.6.0. Log2 of the fold-change (Log2FC) was calculated by HPRT-deficient FPKM—wild-type FPKM. Fold-Change is calculated as 2^(log2FC). A Fisher’s exact test was used to estimate the statistical significance between the two groups (HPRT-deficient and wild-type). The differentially expressed genes were filtered using a fold-change cutoff ≥ 1.5 or ≤0.66, and *p* value ≤ 0.05.

### 2.10. Gene Ontology Analysis

Gene Ontology (GO) describes genes, gene products, and their attributes using a unified and controlled vocabulary (GO terms) across all species [41,42]. The GO annotations included defined terms that describe the pathways and larger processes to which that gene product’s activity contributes, known as the Biological Process (BP). We use the PANTHER Bioinformatics tool for the GO enrichment analysis to investigate whether specific BP GO terms are more likely to be associated with the differentially expressed genes in undifferentiated or differentiated HPRT-deficient cells compared with wild-type cells [43]. For each BP GO term, we analyzed the number of differentially expressed genes associated with the term (count) and the number of background population genes associated with the term (size). The total number of differentially expressed genes (Num Int) and the total number of background population genes (Num Total) were also obtained. The fold enrichment value of the term equals (Count/Size)/(num Int/num Total). The Mann–Whitney test was used to estimate the statistical significance of enrichment of terms between the two groups. The *p*-value denotes the significance of BP GO term enrichment in the differentially expressed gene list, and we selected a *p* value ≤ 0.05.

## 3. Results

### 3.1. Generation of Pluripotent HPRT-Deficient Cells

**Figure 1:**
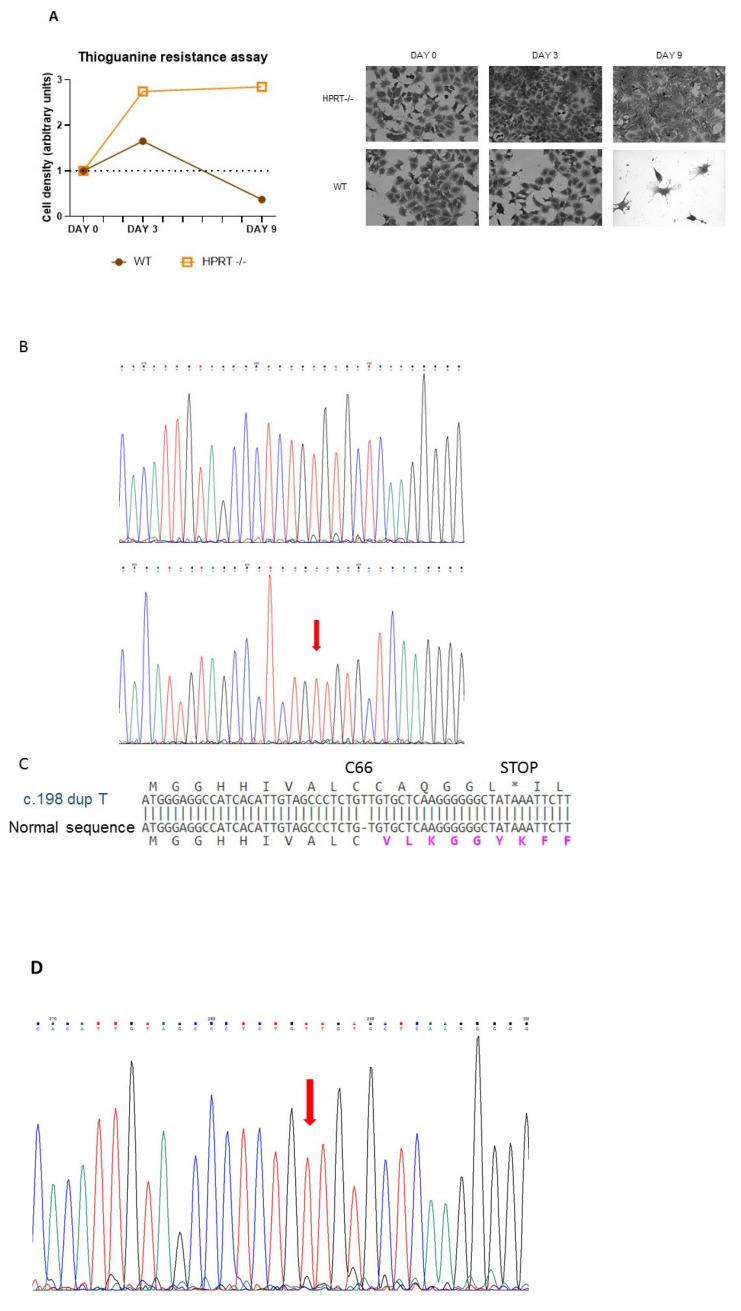

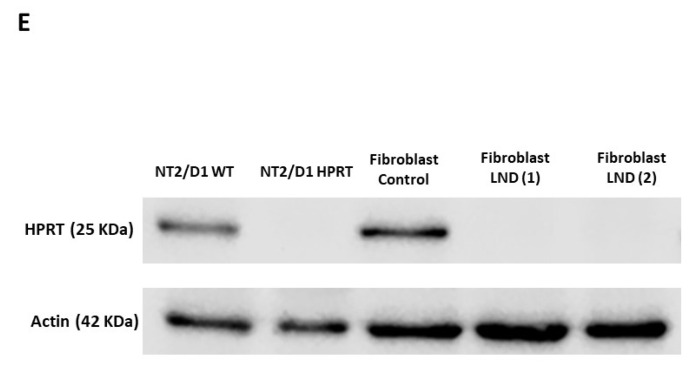
Characterization of HPRT-deficient pluripotent human embryonic cell line NT2/D1 generated by CRISPR-Cas9. (**A**) Selection of HPRT-deficient edited cells in medium with 6-thioguanine 30 μM for 10 days. (**B**) Chromatogram of sequenced DNA obtained from wild-type and 6-thioguanine-resistant HPRT-deficient edited cells showing a duplication of thymine in exon 3 of the HPRT1 gene after position c.198 (NM_000194.2) (c.198dupT; p.Val67CysfsTer7) in HPRT-deficient cells. (**C**) Effect of c.198dupT duplication in HPRT protein. This duplication, caused by a CRISPR-Cas9 T insertion, alters the reading frame after C66 and results in a stop codon at position 73 in the HPRT transcript. * stop codon. (**D**) Chromatogram of sequenced *HPRT1* cDNA obtained from reverse transcribed and amplified RNA from 6-thioguanine-resistant HPRT-deficient edited cells, showing the c.198dupT duplication. (**E**) Western blot of cell lysate from HPRT-deficient edited cells and LND fibroblasts.

We generated an HPRT-deficient line from the pluripotent human embryonic cell line NT2/D1 by knocking out the *HPRT1* gene using gene editing by CRISPR-Cas9 with AGCCCCCCUUGAGCACACAG as a guide sequence and AGG as the PAM. This guide sequence matches nucleotides c.195–214 of the HPRT1 coding sequence (NM_000194.2). The modified cells were selected by culture with 6-thioguanine 30 μM for several weeks. Figure 1A shows how wild-type and 6-thioguanine-resistant cells grow for 10 days in regular DMEM containing 30 µM 6-thioguanine. Wild-type cells died due to their inability to survive in this medium, while most of the edited cells survived and proliferated. In sequenced DNA obtained from 6-thioguanine-resistant HPRT-deficient edited cells, a duplication of thymine in exon 3 of the *HPRT1* gene after position c.198 (NM_000194.2) (c.198dupT; p.Val67CysfsTer7) was observed (Figure 1B). This duplication alters the reading frame after C66 and results in a stop codon at position 73 in the *HPRT1* transcript (Figure 1C). In the cell lysate obtained from 6-thioguanine-resistant HPRT-deficient edited cells, HPRT activity was undetectable (<0.01 nmol/h/mg protein; n = 6), whereas in wild-type cells it was 217 ± 58 nmol/h/mg protein (n = 6). Adenine phosphoribosyltransferase (APRT) activity, simultaneously assayed, was significantly higher in HPRT-deficient cells as described in other HPRT deficient cell types (609 ± 163 nmol/h/mg protein in HPRT-deficient cells vs. 369 ± 117 nmol/h/mg protein in wild-type cells; *p* = 0.0149, n = 6) Moreover, in the culture medium we found a significantly higher excretion of hypoxanthine in HPRT-deficient cells (252.3 ± 18.6 nmol/mg protein) compared to wild-type cells (21.7 ± 18.6 nmol/mg protein; *p* = 0.0019, n = 6) and xanthine (164.3 ± 89.4 nmol/mg protein vs 59.6 ± 26.2 nmol/mg protein; *p* = 0.0203, n = 6). RNA was obtained from wild-type and 6-thioguanine-resistant HPRT-deficient edited cells and reverse transcribed. *HPRT1* cDNA was amplified by PCR and sequenced, and the c.198dupT duplication was found in the cDNA sequence of 6-thioguanine-resistant HPRT-deficient edited cells (Figure 1D). *HPRT1* mRNA expression, measured as *HPRT1*/*ACTB* ratio, was significantly decreased in 6-thioguanine-resistant HPRT-deficient edited cells compared with wild-type cells (0.025 ± 0.002 vs 0.233 ± 0.036; *p* < 0.001, n = 6). As shown in Figure 1E, HPRT-deficient edited cells do not express HPRT protein, similarly to human fibroblasts obtained from LND patients. Taken together, these results support the suitability of the model for studying LND.

### 3.2. Real-Time Quantitative PCR Array of Selected Genes Related to Neuronal Differentiation

We analyzed by qPCR the expression of 42 genes: 37 genes related to neural differentiation selected from the literature published on the subject, the *HPRT1* gene, and 4 housekeeping genes for normalization (Supplementary Table S1). Total RNA was isolated from three wild-type and three HPRT-deficient cell cultures and analyzed by qPCR, as described in the Materials and Methods Section.

#### 3.2.1. Wild-Type Versus HPRT-Deficient Cells

**Figure 2:**
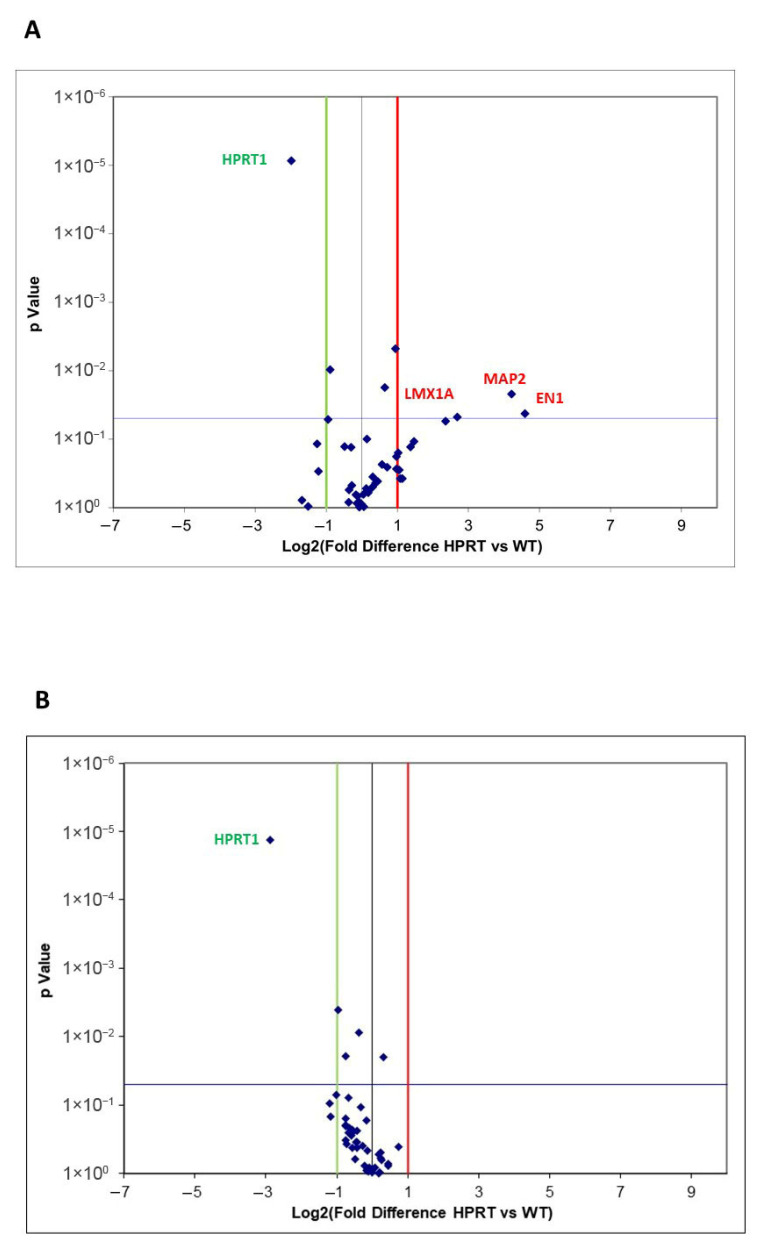
Volcano Plot of differentially expressed genes by real-time quantitative PCR array of selected genes related to neuronal differentiation in HPRT-deficient and wild-type NTD2/D1 cells. (**A**) Volcano Plot of differentially expressed genes in undifferentiated HPRT-deficient cells (HPRT) versus wild-type (WT) cells. (**B**) Volcano Plot of differentially expressed genes in differentiated HPRT-deficient cells (HPRT) versus wild-type (WT) cells. EV values ≤ −2.0 are represented by a green line (decreased expression) and EV values ≥ 2.0 by a red line (increased expression), and a *p* value < 0.05 (considered significant) as a blue line.

Differentially expressed genes in HPRT-deficient versus wild-type cells are presented as a Volcano Plot in Figure 2A. EV values ≤ −2.0 are represented by a green line (decreased expression) and EV values ≥ 2.0 by a red line (increased expression), and a *p* value < 0.05 (considered significant) is represented as a blue line. Individual values of EV and *p* values for each analyzed gene are presented in Supplementary Table S2. As expected, we found a significantly decreased expression of *HPRT1* in HPRT-deficient cells compared with wild-type cells (EV −3.96, *p* = 0.0001). A significantly higher expression of engrailed homeobox 1 (*EN1*; EV 24.21, *p* 0.042), microtubule-associated protein 2 (*MAP2*; EV 18.5, *p* 0.022), and LIM homeobox transcription factor 1 alpha (*LMX1A*; EV 6.43, *p* 0.048) was observed in HPRT-deficient cells compared to wild-type cells.

#### 3.2.2. Differentiated Wild-Type Versus Differentiated HPRT-Deficient Cells

Gene expression results are presented as a Volcano Plot of differentially expressed genes in RA-differentiated HPRT-deficient versus wild-type cells (Figure 2B). EV values ≤ −2.0 are represented by a green line (decreased expression) and EV values ≥ 2.0 by a red line (increased expression), and a *p* value < 0.05 (considered significant) as a blue line. Only a significantly decreased expression of *HPRT1* was found in HPRT-deficient cells compared to wild-type cells (EV −7.36, *p* < 0.0001). Individual EV and *p* values for each analyzed gene are presented in Supplementary Table S2.

### 3.3. Differential Global Gene Expression by RNAseq

#### 3.3.1. Wild-Type Versus HPRT-Deficient Cells

**Figure 3:**
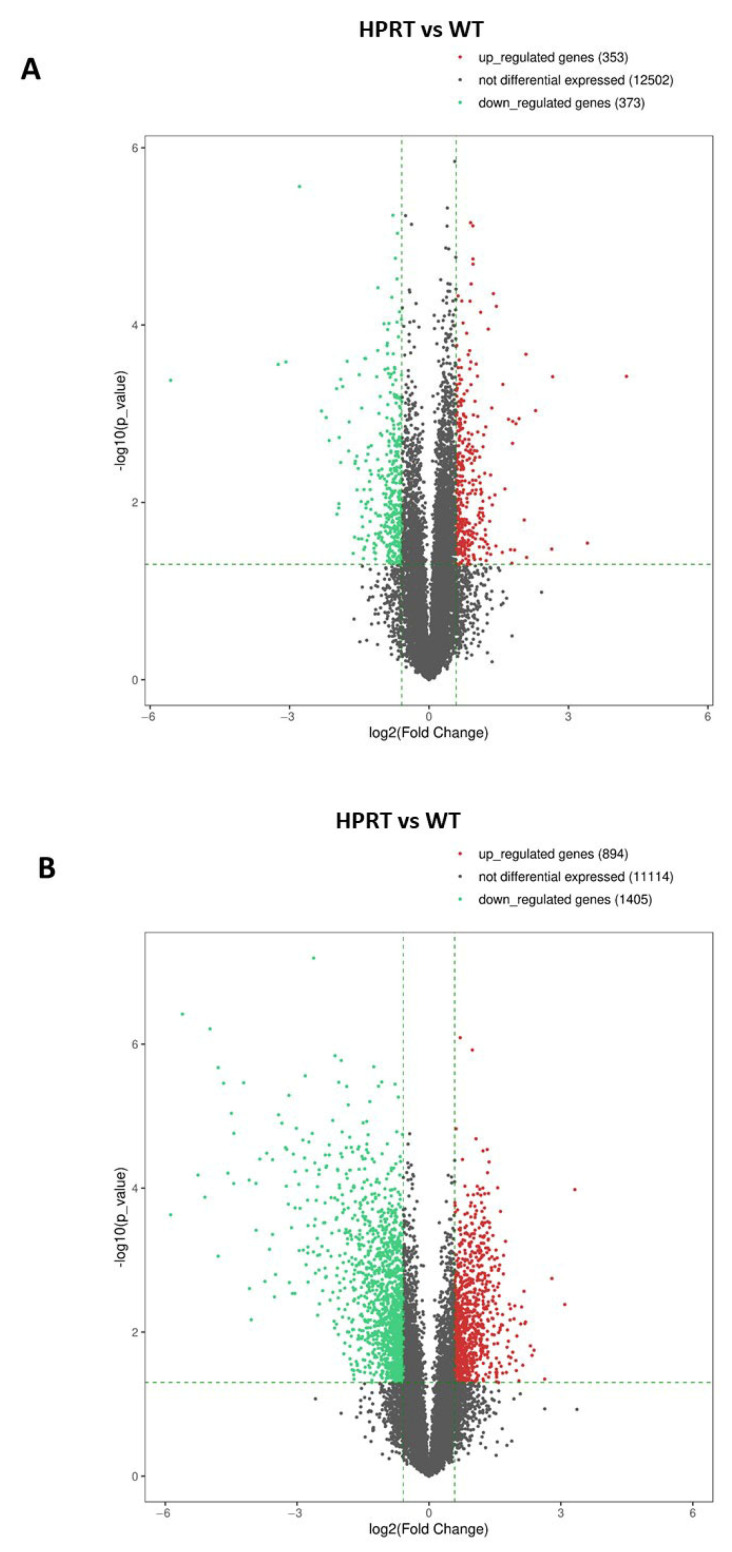
Volcano Plot of differentially expressed genes by RNAseq in HPRT-deficient and wild-type NTD2/D1 cells. (**A**) Volcano Plot of differentially expressed genes in undifferentiated HPRT-deficient cells versus undifferentiated wild-type cells. (**B**) Volcano Plot of differentially expressed genes in differentiated HPRT-deficient cells versus differentiated wild-type cells. EV values ≥ 1.5 (LogFC ≥ 0.585) and ≤−1.5 (Log FC ≤ −0.585) (vertical dotted lines in the Volcano Plot) with a *p* < 0.05 (horizontal dotted line in the Volcano Plot) were considered significant. Red dots: upregulated genes. Black dots: non-differentially expressed genes. Green dots: downregulated genes.

RNAseq analysis was performed in three samples from HPRT-deficient cells and three samples from wild-type cells. A Volcano Plot of differentially expressed genes showed 353 upregulated genes, 12,502 non-differentially expressed genes, and 373 downregulated genes in HPRT-deficient cells compared with wild-type cells (Figure 3A). EV values ≥ 1.5 (LogFC ≥ 0.585) and ≤−1.5 (Log FC ≤ −0.585) (vertical dotted lines in the Volcano Plot) with a *p* < 0.05 (horizontal dotted line in the Volcano Plot) were considered significant. The most significant differentially expressed genes are shown in Table 1 and Table 2. Table 1 shows differentially downregulated genes in HPRT-deficient cells with a fold-change cutoff of 0.33. *DPPA3* (developmental pluripotency-associated 3), also known as *STELLA*, was the most downregulated gene (fold change 0.021; *p* = 0.0004). The genes *CCN2* (also known as *CTGF*) and *CCN1* (also known as *CYRG61*) that codify for growth factors that regulate cell adhesion were clearly downregulated (fold change 0.105 and 0.118, respectively, *p* < 0.0003). As expected, *HPRT1* was one of the most downregulated genes (fold-change 0.144; *p* < 0.0001). *CFAP95* (cilia and flagella-associated protein 95), also known as *C9orf135*, is another pluripotent gene downregulated (fold change 0.302; *p* = 0.0026). *NNMT* (nicotinamide N-methyltransferase) was also downregulated in HPRT-deficient cells (fold-change 0.253; *p* 0.013). Table 2 shows differentially upregulated genes in HPRT-deficient cells with a fold-change cutoff of 3.0. Collagen type III alpha 1 (*COL3A1*, fold-change 19.0, *p* < 0.0004), collagen type II alpha 1 (*COL2A1*, fold-change 6.2, *p* = 0.03), and *TMEM189-UBE2V1* (fold-change 10.6, *p* < 0.03) were the most upregulated genes. The increased expression of *EN1*, *MAP2*, and *LMX1A* found by qPCR array in HPRT-deficient cells compared to wild-type cells was not significant by RNAseq (*EN1*; 1.13-fold, *p* 0.407; *MAP2*; 1.45-fold, *p* 0.161; *LMX1A*; 2.56-fold, *p* 0.158).

#### 3.3.2. Differentiated Wild-Type Versus Differentiated HPRT-Deficient Cells

RNAseq analysis was performed in three samples from HPRT-deficient cells and three samples from wild-type cells. A Volcano Plot of differentially expressed genes showed 894 upregulated genes, 11,114 non-differentially expressed genes, and 1405 downregulated genes in HPRT-deficient cells compared to wild-type cells (Figure 3B). EV values ≥ 1.5 (LogFC ≥ 0.585) and ≤−1.5 (Log FC ≤ −0.585) (vertical dotted lines in the Volcano Plot) with a *p* < 0.05 (horizontal dotted line in the Volcano Plot) were considered significant. In both wild-type and HPRT-deficient cells, differentiation is accompanied by the total or almost total disappearance of the expression of stem cell factor genes as *NANOG* and P*OU5F1* (*OCT4*). In addition, there is an increase in the expression of the differentiation markers *DCX* (Doublecortin) and *SNAP25* (Synaptosome-associated protein) (Supplementary Figure S1). Table 3 and Table 4 show the 20 most differentially regulated genes in HPRT-deficient cells and the values for the *HPRT1* gene. We found a significant decrease in H*PRT1* expression in HPRT-edited cells compared with wild-type cells (fold-change 0.243; *p* < 0.0001). Most downregulated genes in differentiated HPRT-deficient cells are related to interferon-induced or interferon-related genes (*IFI6, IFI44L, IFIT1, ISG15, OAS2, MX1, IFI44, OAS3, IFIT3, OAS1, FPKMCXCL10, IFITM1, USP18*) (Table 3). Similarly to what occurs in undifferentiated cells, a decrease in the expression of the *NNMT* gene was observed (fold-change 0.065; *p* < 0.0001).

Table 4 shows the 20 most differentially upregulated genes in differentiated HPRT-deficient cells, with a fold-change cutoff of 3.7. The gene with the highest upregulation was *SAMD11* (fold change 9.9, *p* = 0.0001), a transcriptional repressor of RNA polymerase II. The *HSPA6* gene, which encodes the chaperone *HSP70B*, was second (fold change 8.5, *p* = 0.004), followed by the *GATA2* gene (fold change 6.9, *p* < 0.002), a transcription factor that regulates embryonic development. Several of the most upregulated genes in differentiated HPRT-deficient cells are related to the homeobox gene family of transcription factors implicated in development. There was a significant decrease in *EN1* expression (fold-change 0.400; *p* 0.0252), and an increased expression of *LMX1A* in differentiated HPRT-deficient cells compared to differentiated wild-type cells (fold-change 2.980; *p* 0.0013). Table 5 shows all homeobox genes differentially up- or downregulated both in differentiated HPRT-deficient cells versus differentiated wild-type cells and in undifferentiated HPRT-deficient cells versus undifferentiated wild-type cells.

We also analyzed the differential expression of genes related to purine metabolism in HPRT-deficient cells versus wild-type cells. With a fold-change cutoff of 1.5, only *HPRT1* was differentially expressed in undifferentiated HPRT-deficient cells. In differentiated cells, we also found a significantly higher expression of *IMPDH2* (fold-change 2.73; *p* < 0.0005) and a decreased expression of *ADA* (fold-change 0.37; *p* < 0.005) in HPRT-deficient cells.

### 3.4. Gene Ontology Enrichment Analysis

#### 3.4.1. Wild-Type Versus HPRT-Deficient Cells

Table 6 and Supplementary Figure S2 show the most significant BP GO terms associated with upregulated and downregulated genes in undifferentiated HPRT-deficient cells compared to wild-type cells. Upregulated genes (Supplementary Figure S2A) are most significantly associated with the BP GO terms *Multicellular organism development*, *System development*, *Anatomical structure development*, *Anatomical structure morphogenesis*, *Developmental process*, *Animal organ development*, *Embryo development*, *Response to stimulus*, and *Tissue development*. Downregulated genes (Supplementary Figure S2B) are most significantly associated with the BP GO terms *Response to stimulus*, *Developmental process*, *Anatomical structure development*, *Regulation of signaling*, *Response to organic substance*, *Regulation of response to stimulus*, *Regulation of cell communication*, *Cell communication*, and *signaling.*

#### 3.4.2. Differentiated Wild-Type Versus Differentiated HPRT-Deficient Cells

The most significant BP GO terms associated with up- and downregulated genes in differentiated HPRT-deficient cells compared to wild-type cells are presented in Table 7 and Supplementary Figure S3. Upregulated genes (Supplementary Figure S3A) are most significantly associated with the BP GO terms *Cytoplasmic translation*, *Regulation of nitrogen compound metabolic process*, *Developmental process*, *Multicellular organism development*, *Anatomical structure development*, *Regulation of RNA metabolic process*, *System development*, *Regulation of DNA-template transcription*, and *Regulation of nucleic acid-template transcription.* Meanwhile, downregulated genes (Supplementary Figure S3B) are most significantly associated with *Response to stimulus*, *Response to organic substance*, *Regulation of response to stimulus*, *Developmental process*, *Immune system process*, *Defense response*, *Biological process involved in interspecies interaction between organisms*, *Anatomical structure development*, and *Cell communication.*

Table 8 and Supplementary Figure S4A,B show the significant BP GO terms related to the nervous system associated with up- and downregulated genes in differentiated HPRT-deficient cells compared with wild-type cells. In both up- and downregulated genes, the most represented terms are *Neurogenesis*, *Neuron differentiation*, and *Neuron development.*

## 4. Discussion

We have generated an HPRT-deficient line from the pluripotent human embryonic cell line NT2/D1 by knocking out the HPRT1 gene using CRISPR-Cas9 gene editing. These HPRT-deficient cells present a T duplication in HPRT1 exon 3 (c.198dupT; p.Val67CysfsTer7), which causes a frame shift and a premature stop codon in the HPRT protein. These cells present a severe decrease in HPRT activity, are resistant to 6-thioguanine toxicity, and excrete a large quantity of xanthine and hypoxanthine into the medium. They also present a decreased expression of *HPRT1* mRNA, and HPRT protein was not detected by Western blot. As such, we can conclude the suitability of the model to study HPRT deficiency and LND.

The human NT2/D1 cell line is a well-established model of neurogenesis. This cell line shows characteristics of pluripotent cells, as expression of *OCT4* and *NANOG* [44]. These cells differentiate along neuro-ectodermal lineages after exposure to RA, developing neuron-like properties [30,31]. Differentiated cells can establish functional synapses and present neuronal electrophysiological properties [30,31,44]. In our laboratory, we have previously employed this model to examine the effect of hypoxanthine and AICAr excess on neural RA-induced differentiation by the changes in the expression of various transcription factor genes [9,45]. As purine alterations in LND fibroblasts depend significantly on the level of folic acid in the culture medium [8], we evaluated the effect of HPRT deficiency on neural development in cells maintained with DMEM containing physiological levels of folic acid. Recently, we have described a new cell culture medium (Plasmax-PV) that contains physiological levels of all nutrients, including vitamins [29]. Future experiments will determine whether NT2/D1 cells can grow and differentiate properly in this new culture medium. To study potential neurodevelopmental alterations in HPRT-deficient NT2/D1 cells, we followed two methodological approaches: a directed qPCR array of genes related to neuronal differentiation, and a global gene expression analysis by RNAseq in both undifferentiated and RA-induced differentiated cells. Both qPCR array and RNAseq (Supplementary Table S2 and Table 1 and Table 3) confirmed the significantly decreased expression of *HPRT1* in HPRT-deficient cells compared to wild-type cells in both undifferentiated and differentiated NT2/D1 cells, thus highlighting the validity of the model and the methodology.

A global gene-expression analysis by RNAseq in undifferentiated cells showed 353 upregulated genes and 373 downregulated genes in HPRT-deficient cells compared to wild-type cells (Figure 3A). Of these, the most downregulated gene in HPRT-deficient cells was *DPPA3*, also known as *STELLA* (Table 1). *DPPA3* is a developmentally regulated gene that is highly expressed in embryonic stem cells, and studies carried out to date indicate that DPPA3 plays a significant role in early mouse embryonic development and affects the differentiation of human embryonic stem cells [46]. Recently, it has been reported that DPPA3 protects UHRF1 and NANOG from degradation in embryonic stem cells [47]. Importantly, UHRF1 is involved in maintaining DNA methylation homeostasis [48]. Another gene downregulated in undifferentiated HPRT-deficient cells is *CFAP95* (Table 1), also known as *C9orf135*, which encodes a membrane protein related to pluripotency in human embryonic stem cells [49]. Analysis of fold-changes between human embryonic stem cells and differentiated cells, by expression profiling of seven microarray data, found that *CFAP95* expression was sharply downregulated during differentiation in all seven studies, along with other known pluripotency genes [49]. Interestingly, C*FAP95* expression is regulated synergistically by P*OU5F1* (*OCT4*) and *SOX2* transcription factors [49], and we have found that both genes are downregulated in HPRT-deficient NT2/D1 cells (Table 5). Other homeobox genes were also differentially expressed in undifferentiated HPRT-deficient cells (Table 5).

It is noteworthy that two of the three genes more differentially downregulated in HPRT-deficient undifferentiated cells (*CNN1* and *CNN2*) codify for growth factors that regulate cell adhesion, and two of the three genes more differentially upregulated codify for collagen proteins (COL3A1 and COL2A1). We have found recently that fibroblasts from LND patients have reduced cell migration compared to controls [29], and it has been reported that LND fibroblasts have higher adhesion than controls [50]. It would be interesting to analyze if there are changes in the composition of the extracellular matrix in HPRT-deficient cells that affect their adhesion and migration properties. This could be relevant, since it has been reported that HPRT knock-out mice have an alteration in the proliferation and migration of developing midbrain dopamine (mDA) neurons [14].

The gene *NNMT* is also downregulated in undifferentiated HPRT-deficient cells (Table 1). This gene encodes the protein nicotinamide N-methyltransferase, an enzyme that catalyzes the methylation of nicotinamide using S-adenosyl methionine as a methyl donor. Importantly, S-adenosyl methionine has been employed as a treatment for self-injurious behavior in LND in an attempt to restore the purine pool by serving as an adenosine donor [51,52].

RA-induced differentiation of wild-type and HPRT-deficient NT2/D1 cells induced similar morphological changes with no obvious differences, although neurite length was not measured. In the differentiated HPRT-deficient NT2/D1 cells, RNAseq showed 894 upregulated genes and 1405 downregulated genes, including *HPRT1*, compared to differentiated wild-type cells (Figure 3B). Several of these dysregulated genes belong to the homeobox gene family of transcription factors implicated in development (26 homeobox genes upregulated and 7 downregulated) (Table 5). These include a significant decrease in Engrailed homeobox 1 (*EN1*), together with an increased expression of LIM homeobox transcription factor 1 alpha (*LMX1A*). Changes in the expression of *EN1* and L*MX1A* genes in HPRT-deficient cells have previously been described in both mouse and human cultures, but to our knowledge, dysregulation of other genes of the homeobox gene family has not been hitherto reported in LND models. Reported changes in the expression of *EN1* and *LMX1A* genes in HPRT-deficient cells have shown conflicting results. Our results in undifferentiated cells obtained by qPCR array are in agreement with those of Ceballos-Picot et al. [22], who applied microarray methods and quantitative PCR to 10 different HPRT-deficient sublines derived from the mouse MN9D cell line. These authors found significant variability between the different mutant sublines, but they reported consistent increases in the mRNAs for *EN1* and *EN2* and increases in EN proteins overall. They also found overexpression of EN in fibroblasts from patients with LND. However, we were not able to confirm these results with RNAseq. In contrast, Kang et al. [24] reported that *LMX1A* and *EN1* genes were both downregulated in HPRT knockdown SH-SY5Y cells in both the undifferentiated basal state and after differentiation. The reasons for these discrepancies are unknown. Sutcliffe et al. [18] described 6 iPSC lines from LND patients that presented significant heterogeneity among them in RNAseq gene expression studies, with no obvious influence of within-subject or between-subject relationships. So, variability between cell lines, probably including epigenetic changes and different culture conditions, may explain these disparities. In agreement with our results, increased LMX1A expression has been reported in differentiated NT2/D1 cells 18 days after retinoic acid treatment [23]. As we mentioned before, we did not measure neurite length, but Guibinga et al. reported a deficit in neurite outgrowth during differentiation in HPRT-deficient NT2/D1 cells [23].

Homeobox genes were identified in Drosophila melanogaster thanks to mutations that resulted in homeotic transformations of one body segment to another. These genes are characterized by a consensus DNA sequence of 183 base pairs, known as the homeobox sequence, that encodes the homeodomain. Homeobox genes are crucial for the developmental and post-developmental regulation of morphogenesis, patterning, and differentiation [53], and HOX genes comprise the main subset of the homeobox family. At least 17 HOX genes were upregulated in differentiated HPRT-deficient cells, with *HOXB13*, *HOXB8*, H*OXB9*, and *HOXB5* being the most upregulated (Table 5). Most HOX genes are expressed in the developing vertebrate central nervous system, where they play a role in determining cell fate in hindbrain and spinal cord segments [53]. They also contribute to the establishment of functional neuronal networks. In humans, at least 15 genetic disorders associated with germline mutations in 10 HOX genes have been reported [54]. As mentioned, although to a lesser extent, some homeobox gene family genes are dysregulated in undifferentiated HPRT-deficient cells (Table 5). This fact, along with the dysregulation of *DPPA3* and CF*AP95*, suggests that HPRT-deficient pluripotent cells present abnormalities before they are differentiated that may contribute to alterations of normal development.

Upregulation of other transcription factors was also observed in differentiated HPRT-deficient cells related to wild-type cells (Table 4). *SAMD11* is a transcription repressor of RNA polymerase II and *GATA2*, a zinc-finger transcription factor that seems to be critical in hematopoietic and neurologic development [55]. The gene *HSPA6* (heat shock protein 70 member 6) codifies for a molecular chaperone (HSP70B) involved in a wide variety of cellular processes. Interestingly, HSP70 is involved in the assembly of the purinosome [56], which is clearly increased in fibroblasts from LND patients [57,58].

Regarding genes related to metabolism, we found a decreased expression of the gene *NNMT* in undifferentiated HPRT-deficient cells (fold-change 0.253; *p* < 0.05), which was even more marked in differentiated cells (fold-change 0.065; *p* < 0.0001) (Table 3). We can speculate that preservation of S-adenosyl methionine (an adenosyl donor) may be a cellular adaptation to avoid purine depletion in HPRT-deficient cells. More experiments are necessary to prove this hypothesis. With respect to differential expression of genes related to purine metabolism, besides decreased *HPRT1* expression, only a significantly higher expression of *IMPDH2* and a decreased expression of *ADA* were detected in HPRT-deficient differentiated cells. Although HPRT-deficient cells have an increased de novo purine synthesis [7], we did not detect any significant change in the expression levels of the genes involved (Supplementary Table S3). This is not surprising, since under normal physiological conditions, the expression levels of the enzymes of the de novo pathway usually remain unaltered [59]. Higher activity of the novo purine synthesis in HPRT-deficient cells could be mediated by increased purine synthesis efficiency due to improved purinosome formation. Enzymes involved in the de novo pathway are compartmentalized into a complex known as purinosome that localizes close to the mitochondria, and it has been described that purinosome formation is increased in fibroblasts obtained from LND patients [57,58]. The increased expression of *IMPDH2* in HPRT-deficient differentiated cells is noteworthy. IMPDH2 is the rate-limiting enzyme in the de novo guanine nucleotide biosynthesis and catalyzes the synthesis of XMP from IMP, which will be converted into GMP. Several point mutations in the IMPDH2 gene have been described in patients with dystonia and other neurodevelopmental disorders. According to O’Neill et al. [60], these mutations make the IMPDH2 enzyme insensitive to allosteric inhibition by GTP, thus resulting in increased enzyme activity. Interestingly, IMPDH has been reported to have a non-enzymatic moonlighting role. Mortimer et al. reported that both IMPDH type 1 and IMPDH type 2 are associated with polyribosomes, suggesting that these housekeeping proteins have an unanticipated role in translation regulation [61]. IMPDH also functions as a transcription factor that regulates histone gene expression. Specifically, IMPDH can bind to DNA, particularly CT-rich regions, and repress the transcription of histone genes and other genes like E2f, which is crucial for cell proliferation [62]. Indeed, phosphoribosyl pyrophosphate synthetase, an enzyme involved in the first and rate-limiting step of purine synthesis, has also been identified as a moonlighting protein [63,64]. We also found a decreased expression of the *ADA* gene. It is known that ADA-deficient patients present with typical early-onset severe combined immunodeficiency (ADA-SCID), which may be accompanied by neurologic or behavioral abnormalities [65]. Most downregulated genes in differentiated HPRT-deficient cells are related to interferon-induced or interferon-related genes (Table 3). Interferons are secreted cytokines needed to fight viral infections. They activate a signal transduction cascade that leads to the induction of hundreds of interferon-stimulated genes. Many of the proteins encoded by these genes are related to antiviral defense and stimulation of adaptive immunity [66]. Downregulation of both interferon-induced genes and the *ADA* gene in differentiated HPRT-deficient cells points towards an altered immune response in these cells. In LND patients, a minor impairment of B lymphocyte function was first reported both in vivo and in vitro [67]. However, these abnormalities could not be confirmed by other authors [68].

When we analyzed the biological processes (BPs) associated with differentially expressed genes in HPRT-deficient and wild-type cells by GO enrichment analysis (Table 6 and Table 7), we found that the term “Anatomical structure development” is significantly associated with up- and downregulated genes in both undifferentiated and differentiated cells. This term is defined as “the biological process whose specific outcome is the progression of an anatomical structure from an initial condition to its mature state”. Other biological processes related to development, such as “Developmental process”, “Multicellular organism development”, and “System development”, are also significantly associated with dysregulated genes in both undifferentiated and differentiated HPRT-deficient cells.

If we focus on significant BP GO terms associated with the nervous system, terms related to neurogenesis and neural differentiation are among those that discriminate differentiated HPRT-deficient cells from differentiated wild-type cells (Table 8). A more detailed study of neuronal differentiation in NT2/D1 cells, including quantification and length of their neurites, would be necessary to verify whether the observed differences in gene expression also occur at the morphological level.

## 5. Conclusions

In summary, we have generated an HPRT-deficient pluripotent human embryonic cell line (NT2/D1) and have studied the transcriptome alterations when the cells were cultured at physiological levels of folic acid. Using this model, we found that HPRT-deficient cells present altered expression of genes related to pluripotency in human embryonic stem cells, along with genes from the homeobox gene family. GO enrichment analysis suggests that these alterations are associated with abnormal nervous system development.

A limitation of our study is that the results are restricted to the pluripotent model we have employed, a model that is otherwise widely used and recognized. Neurological symptoms are not reproduced in genetic animal models of LND [13], indicating that rodent brain cells develop alternative metabolic strategies not present in human cells, or some purine-dependent processes are not present during rodent brain development. To delineate potential neurodevelopmental alterations in LND, many investigators have used HPRT-deficient cells. These in vitro studies were performed with cells incubated with nonphysiologically high levels of folic acid, which are present in almost all standard tissue culture media. As we have shown recently, these culture media mask some abnormalities present in fibroblasts obtained from LND patients [8,29]. We believe that the use of physiological levels of folic acid in the culture media represents an improvement in understanding the pathophysiology of LND. However, further studies, employing different cellular models with different neuronal neurotransmitter phenotypes such as dopaminergic, glutamatergic, and cholinergic neurons, will advance the connection between aberrant purine metabolism and the neurological manifestation of LND. These studies should clarify if the effects of HPRT deficiency are limited to the nigrostriatal dopamine pathways or if it is rather a developmental failure that causes dysfunction of widely distributed neural circuits. Interestingly, Bell et al. have shown that HPRT deficiency produces specific alterations in midbrain dopaminergic neuronal progenitor cells but not in cortical neuronal progenitor cells or iPSCs [69]. On the other hand, to our knowledge, the molecular mechanism by which HPRT deficiency causes defective neuronal development is unknown. One possible explanation is the metabolic consequences of HPRT deficiency, such as the toxic effects of hypoxanthine and AICAR in neuronal development or an energy failure due to ATP depletion, which will compromise neuronal growth. Another possibility is that the HPRT protein would have a non-enzymatic moonlighting role. Other metabolic enzymes involved in purine nucleotide synthesis have additional roles in controlling gene expression [61,62,63,64]. Although, to our knowledge, no additional functions of HPRT in the cell or its presence in the nucleus have been described, we cannot rule out a role for the HPRT protein in the control of gene expression.

## Figures and Tables

**Table 1:** Downregulated genes in undifferentiated HPRT-deficient cells compared to wild-type cells. Differentially expressed gene analyses were performed with the R package Ballgown. Fold-change, *p*-value (0.05), and FPKM (0.5 mean in one group) were used to filter differentially expressed genes. Track id: The database name at the gene level. Gene name: The name of the gene. log2FC: if the comparison is test vs control, log2 of the fold-change will be calculated by test FPKM—control FPKM. Fold-Change: 2^(log2FC). *p* value: The *p*-value of the F-statistic for the gene. The *p*-value was set to 1 if any group in the comparison had no replicates. q value: The FDR-adjusted *p*-value. The q value was set to 1 if any group in the comparison had no replicates.

Track id	Gene Name	log2FC	Fold Change	*p* Value	q Value
ENSG00000187569.2_2	DPPA3	−5.560093447	0.02119557	0.000419802	0.040596923
ENSG00000118523.5_2	CCN2	−3.244903704	0.105484015	0.000278163	0.036303633
ENSG00000142871.16_3	CCN1	−3.079243133	0.118319262	0.000260377	0.03573791
ENSG00000165704.14_2	**HPRT1**	−2.786982045	0.144888797	2.73957 × 10^−6^	0.012297513
ENSG00000148677.6_2	ANKRD1	−2.313122967	0.201224382	0.00093275	0.050325601
ENSG00000120937.8_2	NPPB	−2.212740872	0.21572408	0.001104595	0.052000195
ENSG00000197614.10_2	MFAP5	−2.149300526	0.225421882	0.002006182	0.061695869
ENSG00000135046.13_3	ANXA1	−1.987181661	0.252231146	0.000521378	0.043820278
ENSG00000166741.7_3	NNMT	−1.978824709	0.25369646	0.013596552	0.12292049
ENSG00000106366.8_2	SERPINE1	−1.9413966	0.260364273	0.011572679	0.116032592
ENSG00000156265.15_2	MAP3K7CL	−1.938052915	0.26096841	0.01034748	0.111481945
ENSG00000106823.12_3	ECM2	−1.932553968	0.26196501	0.001852116	0.060764564
ENSG00000188015.9_3	S100A3	−1.900994117	0.267758798	0.003546679	0.072840089
ENSG00000101460.12_2	MAP1LC3A	−1.899122452	0.268106397	0.000409214	0.040303113
ENSG00000163347.5_2	CLDN1	−1.857091876	0.276032133	0.000494154	0.042868547
ENSG00000100345.20_2	MYH9	−1.76131462	0.29497925	0.000256178	0.03573791
ENSG00000204711.8_2	CFAP95	−1.727468258	0.30198143	0.002632984	0.065817254
ENSG00000135932.10_2	CAB39	−1.717650144	0.304043543	0.001235067	0.053730422
ENSG00000198380.12_2	GFPT1	−1.631076319	0.322847258	0.037602012	0.180752644
ENSG00000105971.14_2	CAV2	−1.600604271	0.329738838	0.024674901	0.150097079
ENSG00000134531.9_3	EMP1	−1.589696922	0.332241243	0.003645433	0.073941233

**Table 2:** Upregulated genes in undifferentiated HPRT-deficient cells versus wild-type cells. Differentially expressed gene analyses were performed using the R package ballgown. Fold-change, *p* value (0.05), and FPKM (0.5 mean in one group) were used to filter differentially expressed genes. Track id: The database name at the gene level. Gene name: The name of the gene. log2FC: log2 of the fold-change calculated as HPRT-deficient FPKM—wild-type FPKM. Fold-Change: 2^(log2FC). *p* value: The *p*-value of the F-statistic for the gene. The *p*-value was set to 1 if any group in the comparison had no replicates. q value: The FDR-adjusted *p*-value. The q value was set to 1 if any group in the comparison had no replicates.

Track id	Gene Name	log2FC	Fold Change	*p* Value	q Value
ENSG00000168542.14_3	COL3A1	4.247956394	19.00038035	0.000379474	0.039430012
ENSG00000124208.16_3	TMEM189-UBE2V1	3.408428068	10.61791114	0.028791507	0.161419507
ENSG00000139219.17_3	COL2A1	2.640248493	6.234390369	0.03364278	0.172691892
ENSG00000125872.7_2	LRRN4	2.293045787	4.900896867	0.000923861	0.050325601
ENSG00000185664.14_4	PMEL	2.101598092	4.291845351	0.041762031	0.189378261
ENSG00000138080.13_2	EMILIN1	2.086381193	4.246814794	0.00021368	0.033201528
ENSG00000185551.14_3	NR2F2	2.049419436	4.139393602	0.015808124	0.128550994
ENSG00000122691.12_3	TWIST1	1.93767715	3.830883493	0.001135438	0.052593016
ENSG00000116132.11_3	PRRX1	1.87077789	3.657297255	0.001296592	0.0538418
ENSG00000178252.17_3	WDR6	1.840542095	3.581445765	0.034485201	0.17438591
ENSG00000106333.12_2	PCOLCE	1.800690899	3.483870262	0.001220195	0.053665168
ENSG00000163349.21_3	HIPK1	1.79732213	3.475744731	0.002155682	0.063582685
ENSG00000158270.11_3	COLEC12	1.783032855	3.441488896	0.048515744	0.201942638
ENSG00000171161.12_2	ZNF672	1.753629373	3.372058052	0.034188418	0.173644775
ENSG00000022556.15_3	NLRP2	1.711161075	3.27424227	0.001155976	0.052593016
ENSG00000255690.2_2	TRIL	1.63287963	3.101314059	0.007051968	0.096262957
ENSG00000136383.6_2	ALPK3	1.590057032	3.010612508	0.000466886	0.041915559
ENSG00000135903.18_2	PAX3	1.577988651	2.98553328	0.036641362	0.178731018
ENSG00000136110.12_2	CNMD	1.449604509	2.731331661	6.13992 × 10^−5^	0.022921908
ENSG00000117114.19_3	ADGRL2	1.439041207	2.711406098	0.031070439	0.165870885
ENSG00000113196.2_2	HAND1	1.418099884	2.672333167	0.008240986	0.102888602

**Table 3:** The twenty most differentially downregulated genes in differentiated HPRT-deficient cells versus differentiated wild-type cells, and the values for the HPRT1 gene. Differentially expressed gene analyses were performed using the R package Ballgown. Fold-change, *p*-value (0.05), and FPKM (0.5 mean in one group) were used to filter differentially expressed genes. Track id: The database name at the gene level. Gene name: The name of the gene. log2FC: if the comparison is test vs control. log2 of the fold-change will be calculated by test FPKM—control FPKM. Fold-Change: 2^(log2FC). *p* value: The *p*-value of the F-statistic for the gene. The *p*-value was set to 1 if any group in the comparison had no replicates. q value: The FDR-adjusted *p*-value. The q value was set to 1 if any group in the comparison had no replicates.

Track id	Gene Name	log2FC	Fold Change	*p* Value	q Value
ENSG00000126709.14_2	IFI6	−5.878845546	0.016993825	0.000233723	0.014261355
ENSG00000137959.15_3	IFI44L	−5.608066648	0.020502354	3.82253 × 10^−7^	0.002122525
ENSG00000185745.9_2	IFIT1	−5.254460452	0.026196893	6.57308 × 10^−5^	0.00935849
ENSG00000187608.8_4	ISG15	−5.097947283	0.029198797	0.000133271	0.011568582
ENSG00000111335.12_2	OAS2	−4.980723077	0.031670357	6.11792 × 10^−7^	0.002547809
ENSG00000157601.13_3	MX1	−4.796144819	0.035992876	2.11695 × 10^−6^	0.00320583
ENSG00000137965.10_2	IFI44	−4.795653196	0.036005143	0.00088056	0.02125452
ENSG00000111331.12_2	OAS3	−4.672670264	0.039209029	3.48892 × 10^−6^	0.00339028
ENSG00000119917.13_2	IFIT3	−4.576066362	0.041924391	6.18918 × 10^−5^	0.009288229
ENSG00000169429.10_2	CXCL8	−4.495950675	0.044318391	9.12997 × 10^−6^	0.005849499
ENSG00000089127.12_3	OAS1	−4.442799771	0.045981592	8.61343 × 10^−5^	0.01017607
ENSG00000205413.7_3	SAMD9	−4.439354828	0.046091521	1.7325 × 10^−5^	0.007195438
ENSG00000197632.8_3	SERPINB2	−4.215735621	0.053819186	3.44386 × 10^−6^	0.00339028
ENSG00000169245.5_2	CXCL10	−4.091454118	0.058661017	7.72479 × 10^−5^	0.009748454
ENSG00000173391.8_3	OLR1	−4.085638109	0.058897977	0.002476522	0.034021507
ENSG00000185885.15_3	IFITM1	−4.045497159	0.060559741	0.006731331	0.05470077
ENSG00000166741.7_3	NNMT	−3.934604298	0.065398244	8.57421 × 10^−5^	0.01017607
ENSG00000163739.4_2	CXCL1	−3.93287656	0.06547661	0.000384192	0.016613744
ENSG00000184979.9_2	USP18	−3.849155456	0.0693887	3.94644 × 10^−5^	0.008176704
ENSG00000125730.16_3	C3	−3.733039094	0.075204401	0.00197069	0.030365633
ENSG00000165704.14_2	**HPRT1**	−2.039546227	0.243240232	8.9983 × 10^−5^	0.010363433

**Table 4:** The twenty most differentially upregulated genes in differentiated HPRT-deficient cells versus differentiated wild-type cells. Differentially expressed gene analyses were performed using the R package Ballgown. Fold-change, *p*-value (0.05), and FPKM (0.5 mean in one group) were used to filter differentially expressed genes. Track id: The database name at the gene level. Gene name: The name of the gene. log2FC: if the comparison is test vs control. log2 of the fold change will be calculated by test FPKM—control FPKM. Fold-Change: 2^(log2FC). *p* value: The *p*-value of the F-statistic for the gene. The *p*-value was set to 1 if any group in the comparison had no replicates. q value: The FDR-adjusted *p*-value. The q value was set to 1 if any group in the comparison had no replicates.

Track id	Gene Name	log2FC	Fold Change	*p* Value	q Value
ENSG00000187634.11_4	SAMD11	3.317151755	9.966947676	0.000104619	0.010578182
ENSG00000173110.7_3	HSPA6	3.089883973	8.514276679	0.004120717	0.042794832
ENSG00000179348.11_3	GATA2	2.793936011	6.935192898	0.001796619	0.028994304
ENSG00000159184.7_2	HOXB13	2.63230707	6.200166978	0.044819433	0.145877709
ENSG00000118432.12_3	CNR1	2.389582626	5.24005744	0.01782143	0.088564849
ENSG00000170689.9_3	HOXB9	2.343247752	5.074436947	0.020976437	0.09589378
ENSG00000158164.6_2	TMSB15A	2.307040628	4.948669273	0.015467662	0.082319589
ENSG00000159182.4_2	PRAC1	2.197080343	4.585504101	0.007224322	0.056393045
ENSG00000111341.9_2	MGP	2.182372513	4.538993785	0.007587234	0.057716822
ENSG00000196361.9_3	ELAVL3	2.162321548	4.47634598	0.002707677	0.035265434
ENSG00000124194.16_3	GDAP1L1	2.132951579	4.386139141	0.028791242	0.114518746
ENSG00000120068.6_3	HOXB8	2.107315751	4.308888453	0.004464332	0.044718486
ENSG00000120075.5_3	HOXB5	2.087021759	4.248700824	0.007689973	0.058020496
ENSG00000162188.5_2	GNG3	2.045937	4.129413815	0.047246979	0.150407077
ENSG00000185559.13_2	DLK1	1.998043763	3.994579836	0.011308426	0.069531367
ENSG00000015592.16_2	STMN4	1.986280174	3.962140869	0.034155545	0.125871793
ENSG00000244242.1_3	IFITM10	1.975606839	3.932936354	0.024756046	0.1053043
ENSG00000142694.6_2	EVA1B	1.952401525	3.870182302	0.004311137	0.043861073
ENSG00000108511.9_3	HOXB6	1.933700212	3.820337809	0.006540761	0.053992073
ENSG00000102924.11_2	CBLN1	1.903898543	3.742230812	0.018988379	0.091299955

**Table 5:** Homeobox genes differentially regulated in differentiated HPRT-deficient cells versus differentiated wild-type cells and in undifferentiated HPRT-deficient cells versus undifferentiated wild-type cells. Differentially expressed gene analyses were performed using the R package Ballgown. Fold-change, *p*-value (0.05), and FPKM (0.5 mean in one group) were used to filter differentially expressed genes. Track id: The database name at the gene level. Gene name: The name of the gene. log2FC: if the comparison is test vs control. Log2 of the fold change will be calculated by test FPKM—control FPKM. Fold change: 2^(log2FC). *p* value: The *p*-value of the F-statistic for the gene. The *p*-value was set to 1 if any group in the comparison had no replicates. q value: The FDR-adjusted *p*-value. The q value was set to 1 if any group in the comparison had no replicates.

Differentiated Cells					
Track id	Gene Name	log2FC	Fold Change	*p* Value	q Value
ENSG00000159184.7_2	HOXB13	2.63230707	6.200166978	0.044819433	0.145877709
ENSG00000170689.9_3	HOXB9	2.343247752	5.074436947	0.020976437	0.09589378
ENSG00000120068.6_3	HOXB8	2.107315751	4.308888453	0.004464332	0.044718486
ENSG00000120075.5_3	HOXB5	2.087021759	4.248700824	0.007689973	0.058020496
ENSG00000108511.9_3	HOXB6	1.933700212	3.820337809	0.006540761	0.053992073
ENSG00000128645.14_3	HOXD1	1.696198455	3.240459616	0.00085944	0.021008689
ENSG00000162761.14_2	LMX1A	1.575401297	2.980183774	0.001391974	0.025863001
ENSG00000128709.12_3	HOXD9	1.285888058	2.438320987	0.002395687	0.03350744
ENSG00000164853.8_2	UNCX	1.268550707	2.409194227	0.020622765	0.094951359
ENSG00000185668.7_3	POU3F1	1.262708621	2.399458102	0.000188344	0.013335661
ENSG00000173917.10_2	HOXB2	1.240553358	2.362891456	0.01081035	0.068082724
ENSG00000198353.7_3	HOXC4	1.189794616	2.281202654	0.001179911	0.024068111
ENSG00000111249.13_2	CUX2	1.18968407	2.281027863	0.012392041	0.072717219
ENSG00000120093.11_3	HOXB3	1.15246945	2.22294068	0.002591134	0.034893382
ENSG00000128710.5_3	HOXD10	1.088479998	2.126498737	0.000640547	0.019493284
ENSG00000105996.6_2	HOXA2	1.047701401	2.067233567	0.002503953	0.034301685
ENSG00000182742.5_3	HOXB4	0.965568029	1.952832254	0.001836083	0.029163542
ENSG00000197576.13_3	HOXA4	0.931164381	1.906814341	0.005248627	0.048384965
ENSG00000170166.5_3	HOXD4	0.911411045	1.88088422	0.045232538	0.14662067
ENSG00000215612.7_4	HMX1	0.887549812	1.850031469	0.034230417	0.126013322
ENSG00000121297.6_2	TSHZ3	0.848984055	1.801232052	0.016044223	0.083608498
ENSG00000177045.7_2	SIX5	0.814854123	1.759120271	0.031145206	0.119736175
ENSG00000128714.5_2	HOXD13	0.770195003	1.705500293	0.025529381	0.107248486
ENSG00000128652.11_3	HOXD3	0.748395572	1.679923541	0.005374364	0.048887126
ENSG00000138136.6_2	LBX1	0.679070809	1.601108203	0.009589715	0.064232198
ENSG00000007372.21_4	PAX6	0.597865349	1.513475529	0.013698394	0.077090491
ENSG00000174306.21_3	ZHX3	−0.722305605	0.606128	0.017358037	0.08736421
ENSG00000016082.14_2	ISL1	−0.728438166	0.60355696	0.00146414	0.026395725
ENSG00000163064.6_2	EN1	−0.86515322	0.548988099	0.00161515	0.027390246
ENSG00000168779.19_3	SOX2	−0.958672156	0.514530265	0.000571957	0.01890408
ENSG00000167034.9_2	NKX3-1	−1.164739985	0.446044643	0.00321428	0.03831649
ENSG00000167157.10_2	PRRX2	−1.184994519	0.439826209	0.014110056	0.078479236
ENSG00000204531.17_3	POU5F1	−1.601000772	0.329648227	0.011198978	0.069170405
**Undifferentiated cells**					
**Track id**	**Gene Name**	**log2FC**	**Fold Change**	***p*** **Value**	**q Value**
ENSG00000116132.11_3	PRRX1	1.87077789	3.657297255	0.001296592	0.0538418
ENSG00000135903.18_2	PAX3	1.577988651	2.98553328	0.036641362	0.178731018
ENSG00000173917.10_2	HOXB2	1.144447461	2.2106145	0.011703642	0.116641064
ENSG00000105996.6_2	HOXA2	0.842597953	1.793276503	0.030772937	0.165161471
ENSG00000170577.7_2	SIX2	0.820143793	1.765581959	0.003329635	0.071309576
ENSG00000016082.14_2	ISL1	0.816106138	1.760647552	0.008983186	0.106504497
ENSG00000128645.14_3	HOXD1	0.77142207	1.706951503	0.001941455	0.061046111
ENSG00000128714.5_2	HOXD13	0.693889659	1.617638971	0.00149567	0.057054801
ENSG00000185668.7_3	POU3F1	0.686390153	1.609251878	0.034548416	0.17438591
ENSG00000179981.9_3	TSHZ1	0.680204656	1.602367046	0.033564051	0.172691892
ENSG00000128709.12_3	HOXD9	0.640661693	1.559044052	0.003497545	0.072360604
ENSG00000229544.8_2	NKX1-2	0.627938656	1.545355389	0.014494861	0.125234663
ENSG00000215612.7_4	HMX1	0.591368116	1.506674858	0.000547214	0.044818464
ENSG00000125816.4_2	NKX2-4	−0.687731407	0.620829317	0.002069811	0.061914637

**Table 6:** The most significant BP GO terms associated with up- and downregulated genes in undifferentiated HPRT-deficient cells versus wild-type cells. Fisher’s exact test was used to estimate the statistical significance of enrichment of terms between the two groups. The *p*-value denotes the significance of GO term enrichment in the differentially expressed gene list. ID: GO ID. Term: Name of the GO term. Count: Number of genes associated with the ID. Size: Number of background population genes associated with the term. Num Int: Total number of DE genes. Num Total: Total number of background population genes. *p* value: Fisher’s exact test. Enrichment Score: Enrichment Score value of the term, which equals (−log10(Pvalue)). Fold_Enrichment: Fold Enrichment value of the term, which equals (Count/Size)/(numInt/numTotal). GeneRatio: Gene Ratio value for genes associated with the term, which equals (Count/numInt).

BP GO Terms Associated with Upregulated Genes									
ID	Term	Count	Size	NumInt	NumTotal	*p* Value	Enrichment Score	Fold Enrichment	Gene Ratio
GO:0007275	**Multicellular organism development**	134	4012	328	34,863	2.41257 × 10^−42^	41.61751927	3.550052586	0.408536585
GO:0048731	**System development**	124	3584	328	34,863	3.35951 × 10^−40^	39.47372364	3.677431539	0.37804878
GO:0048856	**Anatomical structure development**	147	5284	328	34,863	7.75032 × 10^−38^	37.11068015	2.95695992	0.448170732
GO:0009653	**Anatomical structure morphogenesis**	96	2241	328	34,863	1.48542 × 10^−37^	36.82814949	4.55323734	0.292682927
GO:0032502	**Developmental process**	152	5896	328	34,863	1.67503 × 10^−35^	34.77597846	2.740166959	0.463414634
GO:0048513	**Animal organ development**	105	2902	328	34,863	6.25251 × 10^−35^	34.20394575	3.845765536	0.320121951
GO:0009790	**Embryo development**	58	1048	328	34,863	8.56326 × 10^−28^	27.06736094	5.882441584	0.176829268
GO:0050896	**Response to stimulus**	169	8433	328	34,863	7.98053 × 10^−27^	26.09796836	2.130078047	0.515243902
GO:0009888	**Tissue development**	72	1770	328	34,863	2.49522 × 10^−26^	25.60289146	4.323646135	0.219512195
**BP GO Terms Associated with Downregulated Genes**									
**ID**	**Term**	**Count**	**Size**	**NumInt**	**NumTotal**	***p*** **value**	**Enrichment Score**	**Fold Enrichment**	**Gene Ratio**
GO:0050896	**Response to stimulus**	196	8433	325	34,863	5.9275 × 10^−44^	43.22712839	2.493189941	0.603076923
GO:0032502	**Developmental process**	146	5896	325	34,863	2.6317 × 10^−32^	31.57976388	2.656297881	0.449230769
GO:0048856	**Anatomical structure development**	131	5284	325	34,863	2.72826 × 10^−28^	27.56411377	2.659438071	0.403076923
GO:0023051	**Regulation of signaling**	100	3633	325	34,863	4.72001 × 10^−24^	23.32605739	2.95267738	0.307692308
GO:0010033	**Response to organic substance**	83	2578	325	34,863	5.51134 × 10^−24^	23.25874279	3.453636092	0.255384615
GO:0048583	**Regulation of response to stimulus**	109	4250	325	34,863	5.59901 × 10^−24^	23.25188883	2.751179729	0.335384615
GO:0010646	**Regulation of cell communication**	99	3639	325	34,863	2.08179 × 10^−23^	22.68156228	2.918330902	0.304615385
GO:0007154	**Cell communication**	123	5357	325	34,863	3.06338 × 10^−23^	22.51379851	2.463002542	0.378461538
GO:0023052	**Signaling**	119	5200	325	34,863	3.14735 × 10^−22^	21.50205514	2.454850296	0.366153846

**Table 7:** The most significant BP GO terms associated with up- and downregulated genes in differentiated HPRT-deficient cells versus differentiated wild-type cells. Fisher’s exact test was used to estimate the statistical significance of enrichment of terms between the two groups. The *p*-value denotes the significance of GO term enrichment in the differentially expressed gene list. ID: GO ID. Term: Name of GO term. Count: Number of genes associated with the ID. Size: Number of background population genes associated with the term. Num Int: Total number of DE genes. Num Total: Total number of background population genes. *p* value: Fisher’s exact test. Enrichment Score: Enrichment Score value of the term, which equals (−log10(Pvalue)). Fold_Enrichment: Fold Enrichment value of the term, which equals (Count/Size)/(numInt/numTotal). GeneRatio: Gene Ratio value genes associated with the term, which equals (Count/numInt).

BP GO Terms Associated with Upregulated Genes									
ID	Term	Count	Size	NumInt	NumTotal	*p* Value	Enrichment Score	Fold Enrichment	Gene Ratio
GO:0002181	**Cytoplasmic translation**	82	124	763	37,582	2.0816 × 10^−108^	107.6815974	32.57223185	0.107470511
GO:0051171	**Regulation of nitrogen compound metabolic process**	307	6046	763	37,582	1.2740 × 10^−58^	57,89482511	2.501068479	0.402359109
GO:0032502	**Developmental process**	284	5851	763	37,582	3.48369 × 10^−49^	48.45796012	2.390801899	0.372214941
GO:0007275	**Multicellular organism development**	227	3984	763	37,582	4.84291 × 10^−49^	48.31489359	2.806479522	0.29750983
GO:0048856	**Anatomical structure development**	260	5201	763	37,582	1.55391 × 10^−46^	45.80857371	2.462304986	0.340760157
GO:0051252	**Regulation of RNA metabolic process**	213	3761	763	37,582	3.35767 × 10^−45^	44.4739615	2.78953375	0.279161206
GO:0048731	**System development**	204	3556	763	37,582	6.62305 × 10^−44^	43.17894171	2.825685125	0.267365662
GO:0006355	**Regulation of DNA-templated transcription**	197	3399	763	37,582	7.00027 × 10^−43^	42.15488536	2.854765317	0.25819135
GO:1903506	**Regulation of nucleic acid template transcription**	197	3401	763	37,582	7.63059 × 10^−43^	42,11744152	2.853086537	0.25819134
**BP GO Terms Associated with Downregulated Genes**									
**ID**	**Term**	**Count**	**Size**	**NumInt**	**NumTotal**	***p*** **value**	**Enrichment Score**	**Fold Enrichment**	**Gene Ratio**
GO:0050896	**Response to stimulus**	732	8346	1265	37,582	6.4624 × 10^−172^	171.189604	2.605685903	0.578656126
GO:0010033	**Response to organic substance**	342	2712	1265	37,582	7.5572 × 10^−108^	107.1216417	3.746500402	0.270355731
GO:0048583	**Regulation of response to stimulus**	422	4191	1265	37,582	1.3389 × 10^−102^	101.8732672	2.991466563	0.333596838
GO:0032502	**Developmental process**	499	5851	1265	37,582	2.55294 × 10^−97^	96.59295944	2.533726946	0.394466403
GO:0002376	**Immune system process**	293	2206	1265	37,582	1.58977 × 10^−96^	95.79866673	3.945949065	0.231620553
GO:0006952	**Defense response**	234	1406	1265	37,582	3.95178 × 10^−96^	95.40320743	4.944471744	0.184980237
GO:0044419	**Biological process involved in** **Interspecies interaction between** **organisms**	243	1567	1265	37,582	3.00479 × 10^−93^	92.52218606	4.607089401	0.192094862
GO:0048856	**Anatomical structure development**	458	5201	1265	37,582	3.04307 × 10^−92^	91.51668855	2.616182203	0.362055336
GO:0007154	**Cell communication**	460	5246	1265	37,582	4.31433 × 10^−92^	91.36508615	2.605067064	0.363636364

**Table 8:** Significant nervous system-associated BP GO terms related to up- and downregulated genes in differentiated HPRT-deficient cells versus differentiated wild-type cells. Fisher’s exact test was used to estimate the statistical significance of enrichment of terms between the two groups. The *p*-value denotes the significance of GO term enrichment in the differentially expressed gene list. ID: GO ID. Term: Name of GO term. Count: Number of genes associated with the ID. Size: Number of background population genes associated with the term. Num Int: Total number of DE genes. Num Total: Total number of background population genes. *p* value: Fisher’s exact test. Enrichment Score: Enrichment Score value of the term, which equals (−log10(Pvalue)). Fold_Enrichment: Fold Enrichment value of the term, which equals (Count/Size)/(numInt/numTotal). GeneRatio: Gene Ratio value genes associated with the term, which equals (Count/numInt).

BP GO Terms Associated with Upregulated Genes									
ID	Term	Count	Size	NumInt	NumTotal	*p* Value	Enrichment Score	Fold Enrichment	Gene Ratio
GO:0007399	**Nervous system development**	136	2219	763	37,582	2.91618 × 10^−31^	30.53518564	3.018818178	0.178243775
GO:0022008	**Neurogenesis**	88	1259	763	37,582	5.7406 × 10^−24^	23.24104242	3.442803948	0.115334207
GO:0048699	**Generation of neurons**	79	1097	763	37,582	2.28169 × 10^−22^	21.64174417	3.547119452	0.103538663
GO:0030182	**Neuron differentiation**	76	1027	763	37,582	3.00494 × 10^−22^	21.52216429	3.64500811	0.099606815
GO:0007417	**Central nervous system development**	75	1091	763	37,582	4.18264 × 10^−20^	19.37854917	3.386038276	0.098296199
GO:0007420	**Brain development**	60	832	763	37,582	3.11326 × 10^−17^	16.50678452	3.552084384	0.078636959
GO:0048666	**Neuron development**	58	813	763	37,582	1.74529 × 10^−16^	15.75813215	3.513927511	0.076015727
GO:0061564	**Axón development**	37	388	763	37,582	9.47529 × 10^−15^	14.0234076	4.69705179	0.048492792
GO:0031175	**Neuron projection development**	48	648	763	37,582	1.87305 × 10^−14^	13.72745142	3.648560749	0.062909567
**BP GO Terms Associated with Downregulated Genes**									
**ID**	**Term**	**Count**	**Size**	**NumInt**	**NumTotal**	***p*** **value**	**Enrichment Score**	**Fold Enrichment**	**Gene Ratio**
GO:0007399	**Nervous system development**	173	2219	1265	37,582	2.83609 × 10^−25^	24.54727957	2.316211234	0.136758893
GO:0022008	**Neurogenesis**	117	1259	1265	37,582	3.29975 × 10^−23^	22.48151933	2.760892483	0.092490119
GO:0030182	**Neuron differentiation**	90	1027	1265	37,582	1.80993 × 10^−16^	15.74233731	2.603523059	0.071146245
GO:0048699	**Generation of neurons**	93	1097	1265	37,582	4.41822 × 10^−16^	15.35475299	2.518637607	0.073517787
GO:0048666	**Neuron development**	75	813	1265	37,582	4.53421 × 10^−15^	14.34349797	2.740691043	0.059288538
GO:1901214	**Regulation of neuron death**	42	330	1265	37,582	1.76991 × 10^−13^	12.75204903	3.781157025	0.033201581
GO:0007610	**Behavior**	60	611	1265	37,582	1.8082 × 10^−13^	12.74275385	2.917423003	0.04743083
GO:0031175	**Neuron projection development**	57	648	1265	37,582	5.9181 × 10^−11^	10.22781749	2.613299663	0.045059289
GO:0051960	**Regulation of nervous system development**	45	461	1265	37,582	2.32408 × 10^−10^	9.633748122	2.90001972	0.035573123

## Data Availability

The data that support the findings of this study are available from the corresponding author upon reasonable request.

## Supplementary Materials

The following supporting information can be downloaded at: https://www.mdpi.com/article/10.3390/cells14141105/s1, Table S1: Genes included in the quantitative polymerase chain reaction array designed; Table S2: Individual values of EV (expression variation) and *p* values for each analyzed gene in qPCR array; Table S3: Differentially expression of purine metabolism genes in HPRT deficient cells versus wild type cells. Figure S1. Effect of RA-induced differentiation in wild-type and HPRT-deficient NT2 cells on expression of stem cell factors genes *NANOG* and *POU5F1* (*OCT4*) and differentiation marker genes *DCX* (Doublecortin), and S*NAP25* (Synaptosome-associated protein). Figure S2. Most significant BP GO terms associated with (A) upregulated and (B) downregulated genes in undifferentiated HPRT-deficient cells versus wild-type cells. Figure S3. Most significant BP GO terms associated with (A) upregulated and (B) downregulated genes in differentiated HPRT-deficient cells versus wild-type cells. Figure S4. Enrichment Score for significant nervous system-related BP GO terms associated with dysregulated genes in differentiated HPRT-deficient cells versus wild-type cells.
