# Supplementary material for: Gene Expression Analysis of HPRT-Deficient Cells Maintained with Physiological Levels of Folic Acid
Source: Cells. 2025 Jul 18;14(14):1105. doi: 10.3390/cells14141105 (PMC12293902; doi:10.3390/cells14141105)
Supplement: Supplementary file 1 [file cells-14-01105-s001.zip › cells-3709030-supplementary.pdf]

SUPPLEMENTARY TABLES AND FIGURES.

Gene Expression Analysis of HPRT-Deficient Cells Maintained with Physiological Levels of Folic Acid

Rosa J. Torres [1,2,*], Gerard Valentines-Casas [3,4], Claudia Cano-Estrada [3,4], Neus Ontiveros [3,4] and José M. López [3,4]

[1] Department of Biochemistry, Hospital La Paz Institute for Health Research (IdiPaz), 28046 Madrid, Spain
[2] Center for Biomedical Network Research on Rare Diseases (CIBERER), ISCIII, 28006 Madrid, Spain
[3] Institut de Neurociències, Universitat Autònoma de Barcelona, 08193 Cerdanyola del Vallès, Barcelona, Spain; gerard.valentines@autonoma.cat (G.V.-C.); claudia.cano@uab.cat (C.C.-E.); neus.ontiveros@uab.cat (N.O.); josemanuel.lopez@uab.cat (J.M.L.)
[4] Unitat de Bioquímica, Departament de Bioquímica i Biologia Molecular, Facultat de Medicina,
Universitat Autònoma de Barcelona, 08193 Cerdanyola del Vallès, Barcelona, Spain
* Correspondence: rosa.torres@salud.madrid.org; Tel.: +34-917277343

Table S1: Genes included in the quantitative polymerase chain reaction array designed.

**Neuronal Differentiation**		
NM_004422.2	DVL2	Dishevelled segment polarity protein 2
NM_004429	EFNB1	Ephrin-B1
NM_001426.3	EN1	Engrailed homeobox 1
NM_001427.3	EN2	Engrailed homeobox 2
NM_177398.3	LMX1A	LIM homeobox transcription factor 1 alpha
NM_002316.3	LMX1B	LIM homeobox transcription factor 1 beta
NM_002374	MAP2	Microtubule-associated protein 2
NM_006160	NEUROD2	Neurogenic differentiation 2
NM_006161	NEUROG1	Neurogenin 1
NM_024019	NEUROG2	Neurogenin 2
NM_005450	NOG	Noggin
NM_006186.3	NR4A2 0 NURR1	Nuclear receptor subfamily 4 group A member 2
NM_005010	NRCAM	Neuronal cell adhesion molecule
NM_013957	NRG1	Neuregulin 1
NM_003873	NRP1	Neuropilin 1
NM_003872	NRP2	Neuropilin 2
NM_002527	NTF3	Neurotrophin 3
NM_005029.3	PITX3	Paired-like homeodomain 3
**Wnt/β-catenin, pathway**		
NM_017412.3	FZD3	Frizzled class receptor 3
NM_002093.3	GSK3B	Glycogen synthase kinase 3 beta
NM_005430	WNT1	Wingless-type MMTV integration site family, member 1
NM_003394.3	WNT10B	Wingless-type MMTV integration site family, member 10
NM_004626	WNT11	Wingless-type MMTV integration site family, member 11
NM_003391	WNT2	Wingless-type MMTV integration site family member 2
NM_033131	WNT3A	Wingless-type MMTV integration site family, member 3A
NM_030761	WNT4	Wingless-type MMTV integration site family, member 4
NM_032642	WNT5B	Wingless-type MMTV integration site family, member 5B
NM_058238	WNT7B	Wingless-type MMTV integration site family, member 7B
NM_058244	WNT8A	Wingless-type MMTV integration site family, member 8A
NM_003393	WNT8B	Wingless-type MMTV integration site family, member 8B
NM_003396.2	WNT9B or 14B	Wingless-type MMTV integration site family, member 9B or 14B
**TGFβ and SHH pathway**		
NM_000193	SHH	Sonic hedgehog
NM_000660	TGFB1	Transforming growth factor, beta 1
NM_003242	TGFBR2	Transforming growth factor, beta receptor II (70/80kDa)
**Other**		
NM_000674	ADORA1	Adenosine A1 receptor
NM_000675	ADORA2A	Adenosine A2a receptor
NM_000794.3	DRD1	Dopamine receptor D1
NM_000795	DRD2	Dopamine receptor D2
NM_000621.4	HTR2A	5-hydroxytryptamine (serotonin) receptor 2A
NM_000872.4	HTR7	5-hydroxytryptamine (serotonin) receptor 7
NM_000964	RARA	Retinoic acid receptor, alpha
NM_000360	TH	Tyrosine hydroxylase
NM_000194	HPRT1	Hypoxanthine guanine phosphoribosyl transferase
NM_001101	ACTB	Actin, beta
NM_053275	RPLPO	Ribosomal protein, large, P0
NM_000181.3	GUSB	Glucuronidase, beta
NM_001172085	TBP	TATA box-binding protein

Table S2: Individual values of EV (expression variation) and p values for each analyzed gene in qPCR array. The expression variation (EV) analysis, based on the "delta delta Cp" (ΔΔCp) calculation method, allows a comparison between different experimental conditions, after normalizing the gene expression results with the selected reference genes (ACTB, RPLPO, GUSB and TBP). A Student's t-test for 2(-ΔΔCp) values was performed comparing the experimental condition and the control sample, and values with cutoff EV ≥2.0 or and with a $p<0.05$ were considered significant.

	**HPRT deficient vs Wild type undifferentiated cells**		**HPRT deficient vs Wild type RA-differentiated cells**	
**Gene**	**EV**	**p value**	**EV**	**p value**
ADORA1	-2,85	0,963	1,16	0,963
EN2	2,12	0,376	1,37	0,761
MAP2	18,56	**0,022**	-1,49	0,416
NRG1	1,24	0,499	-1,48	0,251
TGFB1	-1,22	0,476	-1,55	0,217
WNT3A	1,64	0,258	-1,67	0,157
ADORA2A	-1,86	**0,010**	-1,31	**0,009**
FZD3	1,93	**0,005**	-1,11	0,902
NEUROD2	-1,40	0,129	-1,69	0,197
NRP1	1,96	0,180	-1,34	0,347
TGFBR2	1,97	0,271	-1,51	0,279
WNT4	2,02	0,159	-1,08	0,926
DRD1	-1,12	0,649	-1,34	0,421
GSK3B	-1,08	0,679	1,05	0,803
NEUROG1	1,04	0,973	-1,68	0,202
NRP2	-1,29	0,840	-1,61	0,254
TH	1,48	0,234	-1,01	0,842
WNT5B	-1,94	0,052	-1,15	0,766
DRD2	-1,04	0,849	-1,40	0,610
HTR2A	1,36	0,414	-1,35	0,234
NEUROG2	1,13	0,604	1,17	0,589
NTF3	2,07	0,283	1,14	1,000
WNT1	-1,04	0,932	-2,04	0,070
WNT7B	-1,10	0,863	-1,47	0,227
DVL2	-2,33	0,295	1,17	0,485
HTR7	1,24	0,355	-1,93	**0,004**
NOG	2,21	0,375	-1,60	0,077
PITX3	-1,29	0,552	-1,38	0,347
WNT10B	5,11	0,055	-1,01	0,951
WNT8A	2,76	0,108	-2,28	0,093
EFNB1	-3,21	0,778	1,19	0,629
LMX1A	6,43	**0,048**	-1,69	0,321
NR4A2	1,31	0,435	-1,25	0,106
RARA	-2,40	0,117	1,14	0,525
WNT11	2,59	0,130	-1,69	**0,019**
WNT8B	1,12	0,593	-1,21	0,390
**EN1**	24,21	**0,042**	1,37	0,713
LMX1B	-1,05	0,977	-1,10	0,836
NRCAM	1,57	**0,018**	-1,64	0,364
SHH	1,00	0,891	1,67	0,406
WNT2	-1,06	0,891	-1,07	0,822
WNT9B	1,14	0,570	-2,28	0,145
HPRT1	-3,96	**0,000**	-7,36	**0,000**

Table S3: Differentially expression of purine metabolism genes in HPRT deficient cells versus wild type cells: Differentially expressed gene analyses were performed using the R package Ballgown. log2FC: if the comparison is test vs control. Log2 of the fold change will be calculated by test FPKM – control FPKM. Fold Change: 2^(log2FC). p value: The p-value of the F-statistic for the gene. The p-value was set to 1 if any group in the comparison had no replicate. q value: The FDR adjusted p-value. The q value was set to 1 if any group in the comparison had no replicate.

Undifferenciated cells				
**Gene Name**	**log2FC**	**Fold Change**	**P value**	**Q value**
PRPS1	-0,006690915	0,995372949	0,859799532	0,920218807
**PRPS2**	**0,449181966**	**1,365265907**	**0,000987825**	**0,050501782**
**PPAT**	**0,262910335**	**1,199896806**	**0,019872433**	**0,138107279**
GART	-0,133980862	0,91131337	0,32164042	0,529134419
PFAS	0,604666121	1,520626798	0,052766504	0,210449862
**PAICS**	**0,453724209**	**1,369571142**	**3,45665E-05**	**0,021580679**
ATIC	0,049027875	1,03456757	0,517905906	0,689839322
ADSL	0,230439509	1,173192301	0,054829272	0,214369864
ADSS	-0,399672321	0,758030435	0,206899075	0,415309167
IMPDH1	-0,20617874	0,866830163	0,124564317	0,321146118
**IMPDH2**	**0,322504442**	**1,250499466**	**0,004693013**	**0,081911019**
**GMPS**	**0,085819279**	**1,061290257**	**0,000632515**	**0,046032166**
**HPRT1**	**-2,786982045**	**0,144888797**	**2,73957E-06**	**0,012297513**
APRT	-0,013338973	0,99079674	0,883320283	0,932991508
**PNP**	**0,455287944**	**1,371056424**	**0,043972535**	**0,193979418**
**ADA**	-0,047880188	0,967356663	0,708845035	0,823140143
ITPA	-0,324969976	0,798315	0,079845672	0,256392492
**Differentiated cells**				
**Gene Name**	**log2FC**	**Fold Change**	**P value**	**Q value**
PRPS1	-0,06891719	0,953353266	0,636232788	0,752671386
PRPS2	0,379645477	1,301022108	0,20081634	0,358235018
PPAT	-0,110421925	0,926317115	0,548888636	0,683771081
**GART**	**-0,244035728**	**0,844379976**	**0,048905392**	**0,153363333**
**PFAS**	**0,407080426**	**1,325999679**	**0,006468485**	**0,053846367**
PAICS	-0,057063213	0,961218811	0,866041956	0,916480603
ATIC	0,065906074	1,046742132	0,358319068	0,51831183
ADSL	-0,140353899	0,907296565	0,635676722	0,752280679
ADSS	-0,080163542	0,945950409	0,620917261	0,740249285
IMPDH1	0,617161873	1,533854751	0,11030622	0,249082419
**IMPDH2**	**1,453412503**	**2,738550535**	**0,000454778**	**0,017472845**
GMPS	0,057050141	1,040336423	0,676707005	0,783961701
**HPRT1**	**-2,039546227**	**0,243240232**	**8,9983E-05**	**0,010363433**
APRT	0,540542414	1,454519274	0,142591926	0,289911842
PNP	-0,191253422	0,875844452	0,389522946	0,545541721
**ADA**	**-1,415260482**	**0,374942045**	**0,003357885**	**0,038898227**
**ITPA**	**0,518142229**	**1,43210992**	**0,024770333**	**0,1053043**

FigureS1

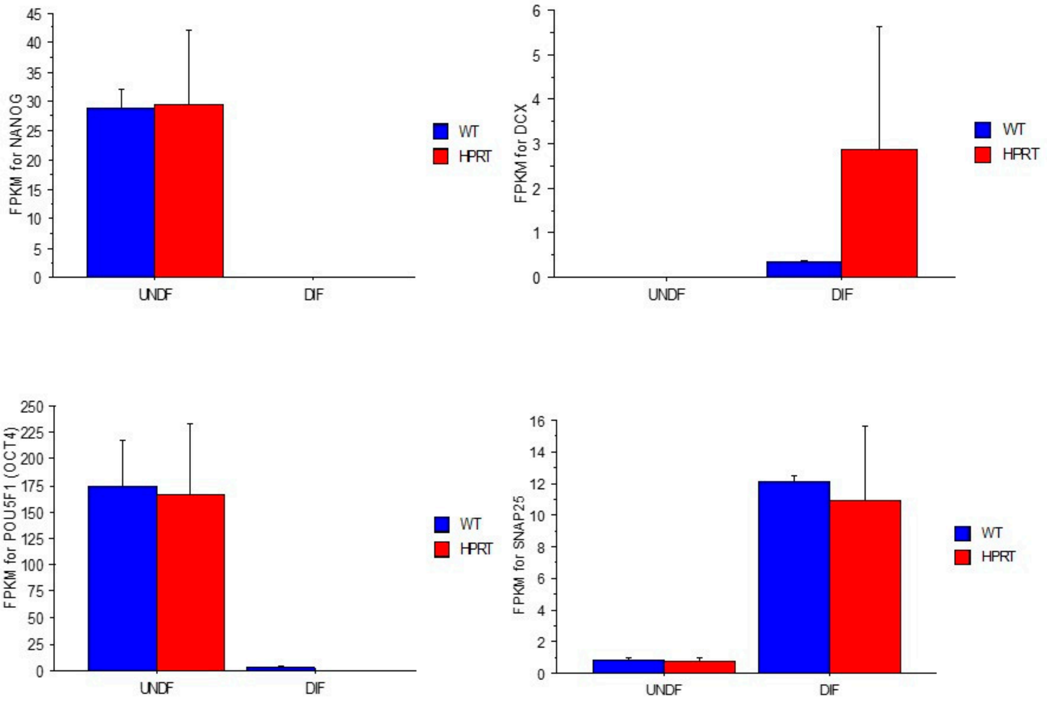

FigureS1. Effect of RA-induced differentiation in wild type and HPRT-deficient NT2 cells on expression of stem cell factors genes NANOG and POU5F1 (OCT4) and differentiation marker genes DCX (Doublecortin), and SNAP25 (Synaptosome associated protein). Gene expression is expressed as mean ± SD of FPKM (Fragments Per Kilobase of transcript per Million mapped reads) of three undifferentiated (UNDF) or differentiated (DIF), wild type (WT, blue bars) or HPRT-deficient (HPRT, red bars) NT2 cells. The FPKM normalizes read count based on gene length and the total number of mapped reads and were calculated using the R package Ballgown (42).

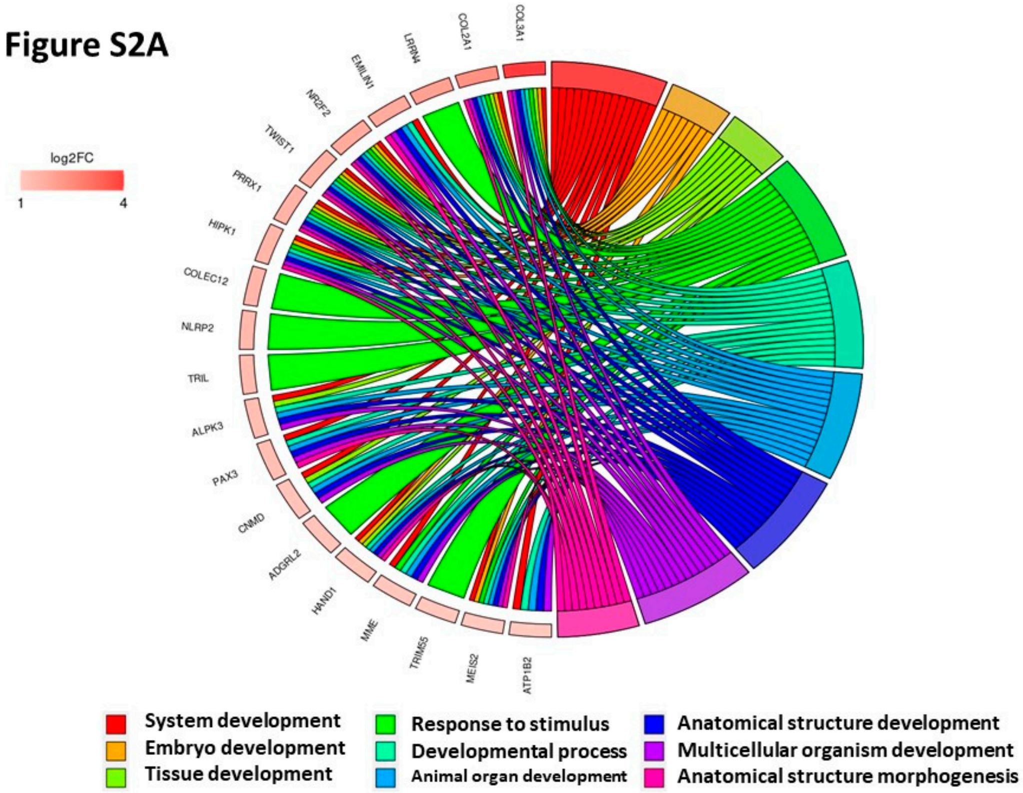

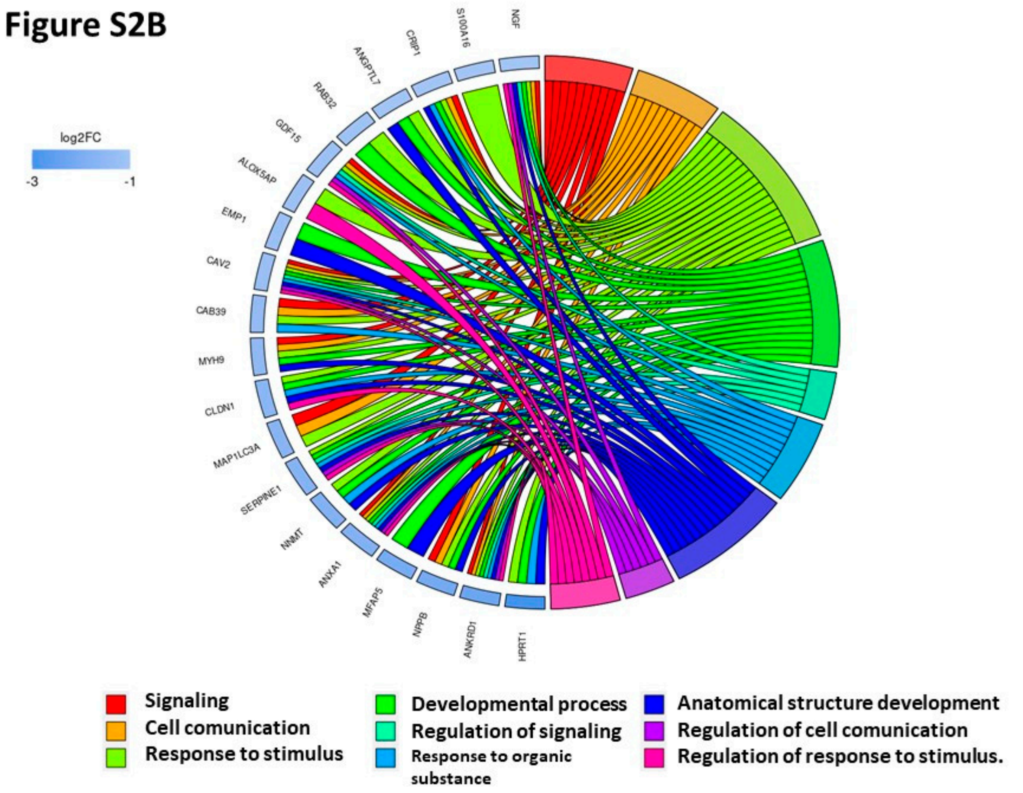

Figure S2. Most significant BP GO terms associated with (A) upregulated and (B) downregulated genes in undifferentiated HPRT-deficient cells versus wild type cells. Circle

plot was constructed: 1. Filtering top 30 significantly differentially expressed genes as candidate genes. 2. Filtering top 9 of all terms (p value) that all candidate genes involved as interest terms. 3. Filtering top 20 of all significantly differentially expressed genes that involved all interest terms as interest genes. 4. Use of interest genes and interest terms to process circle plot.

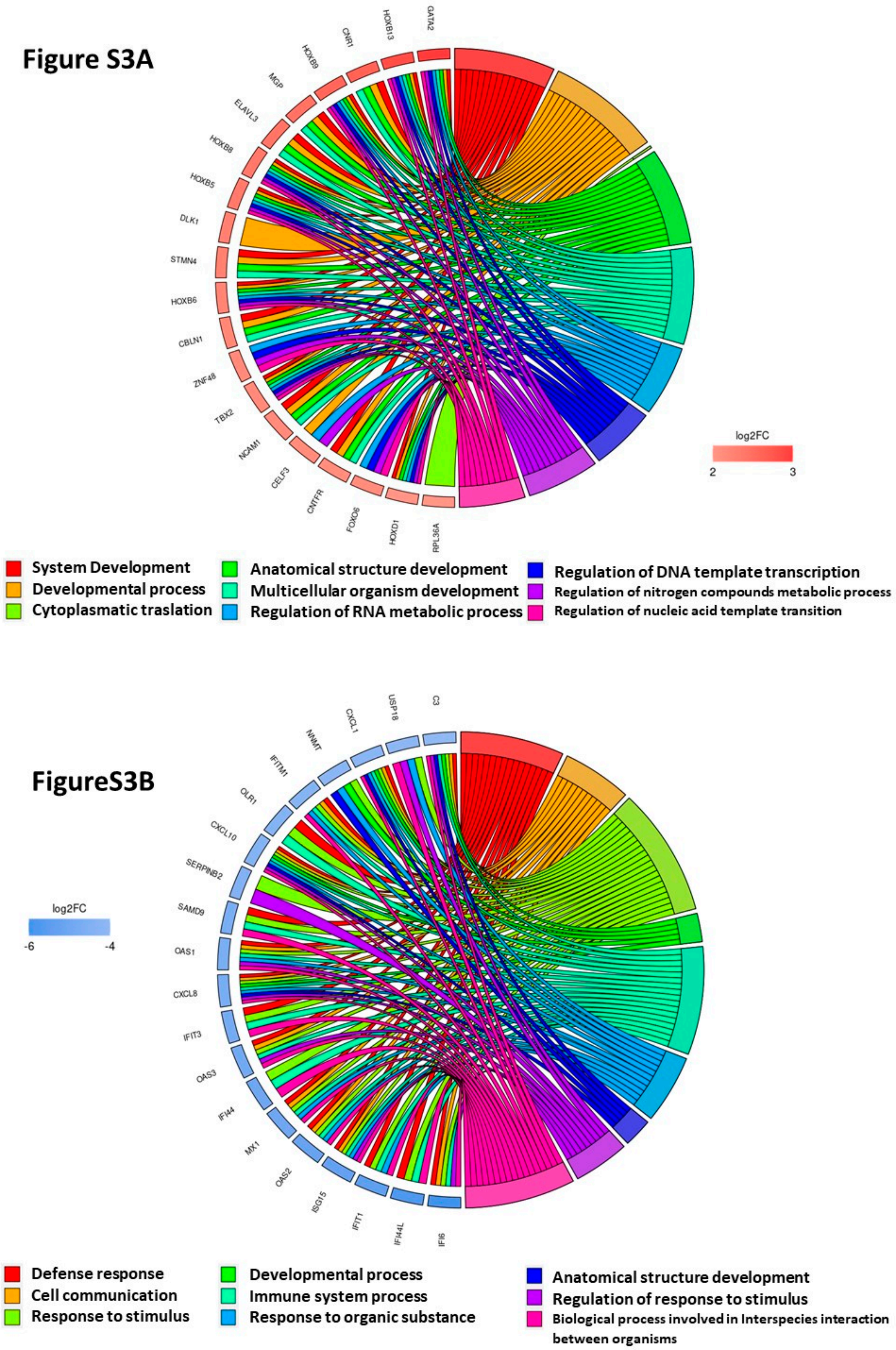

Figure S3. Most significant BP GO terms associated with (A) upregulated and (B) downregulated genes in differentiated HPRT-deficient cells versus wild type cells. Circle

plot was constructed: 1. Filtering top 30 significantly differentially expressed genes as candidate genes. 2. Filtering top 9 of all terms (p value) that all candidate genes involved as interest terms. 3. Filtering top 20 of all significantly differentially expressed genes that involved all interest terms as interest genes. 4. Use of interest genes and interest terms to process circle plot.

Figure S4
A

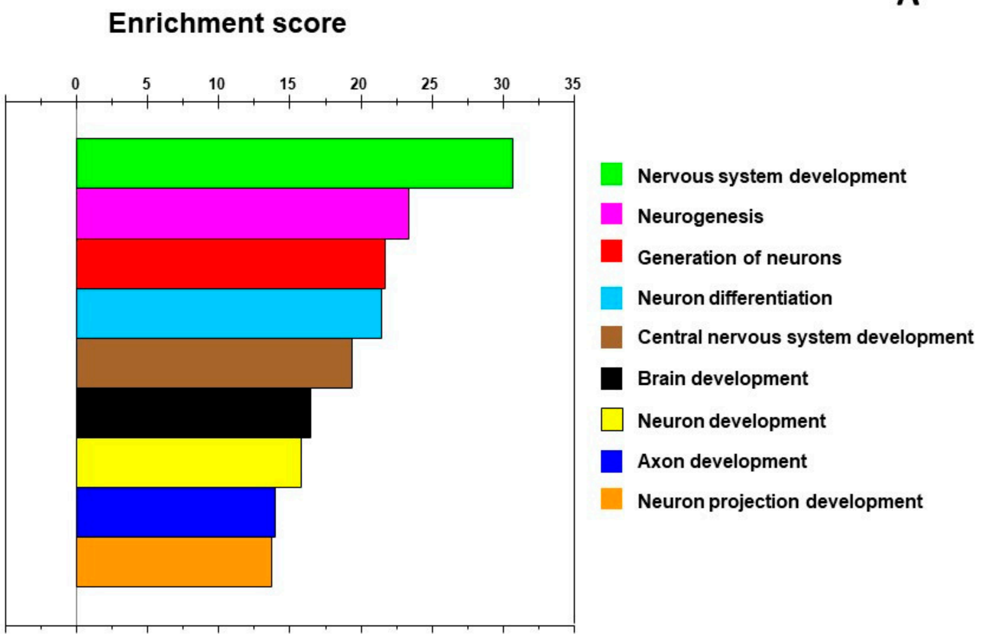

Figure S4
B

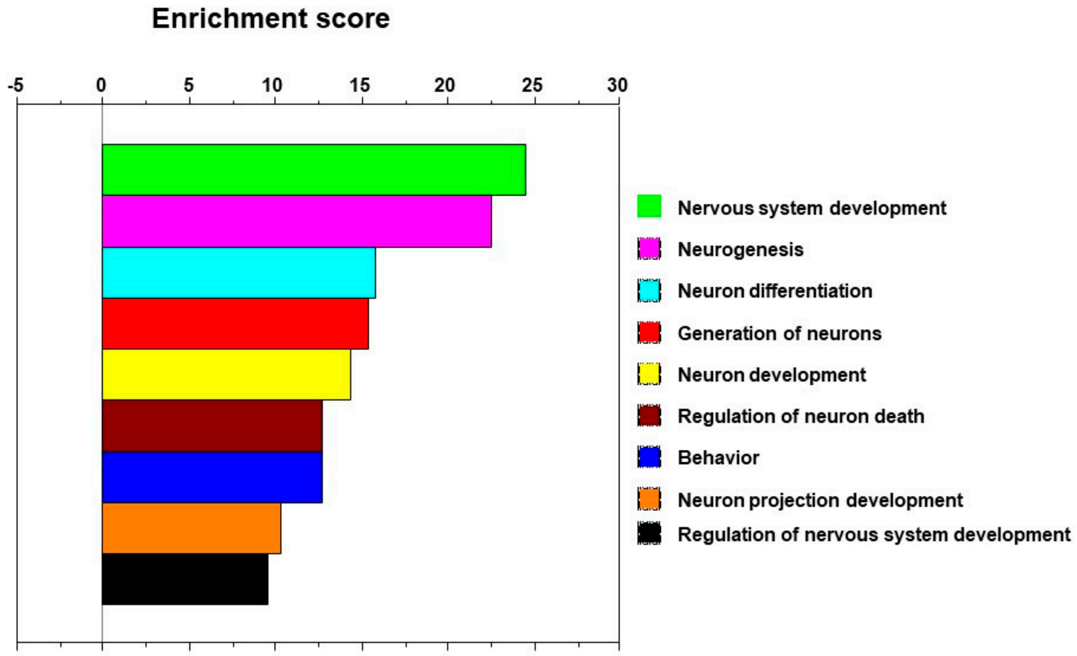

Figure S4. Enrichment Score for significant nervous system-related BP GO terms associated with dysregulated genes in differentiated HPRT-deficient cells versus wild type cells.

Enrichment Score for significant nervous system-related BP GO terms associated with upregulated (A) or downregulated (B) genes in differentiated HPRT-deficient cells versus wild type. Enrichment Score value of the term: -log10$^{(\mathrm{Pvalue})}$.
